# Simulation of alcohol action upon a detailed Purkinje neuron model and a simpler surrogate model that runs >400 times faster

**DOI:** 10.1186/s12868-015-0162-6

**Published:** 2015-04-26

**Authors:** Michael D Forrest

**Affiliations:** Department of Computer Science, University of Warwick, Coventry, West Midlands UK

**Keywords:** Purkinje neuron, Purkinje cell, Purkinje model, Compartmental model, Network model, Reduced neuron model, Simplified, Alcohol, Ethanol, Sodium-potassium pump, Trimodal, Bimodal, Single neuron computation, Cerebellum

## Abstract

**Background:**

An approach to investigate brain function/dysfunction is to simulate neuron circuits on a computer. A problem, however, is that detailed neuron descriptions are computationally expensive and this handicaps the pursuit of realistic network investigations, where many neurons need to be simulated.

**Results:**

We confront this issue; we employ a novel reduction algorithm to produce a 2 compartment model of the cerebellar Purkinje neuron from a previously published, 1089 compartment model. It runs more than 400 times faster and retains the electrical behavior of the full model. So, it is more suitable for inclusion in large network models, where computational power is a limiting issue. We show the utility of this reduced model by demonstrating that it can replicate the full model’s response to alcohol, which can in turn reproduce experimental recordings from Purkinje neurons following alcohol application.

**Conclusions:**

We show that alcohol may modulate Purkinje neuron firing by an inhibition of their sodium-potassium pumps. We suggest that this action, upon cerebellar Purkinje neurons, is how alcohol ingestion can corrupt motor co-ordination. In this way, we relate events on the molecular scale to the level of behavior.

## Background

At present, there are two minimally interacting levels of investigation in computational neuroscience. In the first, researchers use Hodgkin-Huxley models of currents [1] and compartmental/cable modeling of dendrites [2,3] to assemble detailed single neuron descriptions. In the second, researchers study neural circuits and find it useful to represent each neuron and synapse as simply as possible, ignoring much of the biological detail. This demarcation is principally because the computational complexity of the former is not conducive to the scaling of the latter. This issue is unfortunate as there is some evidence that the richness of biophysical properties on the single neuron scale can supply mechanisms that serve as the building blocks for network dynamics [4]. There is a great potential for bridging the gap between these two levels of enquiry, using network models with neurons of intermediate biological fidelity and moderate computational complexity. These studies can unify cellular and networks studies and identify how distinctive single neuron behaviors are important to network and system function.

*In vitro*, within cerebellar slices, Purkinje neurons can spontaneously fire action potentials in a repeating trimodal pattern that consists of tonic spiking, bursting and quiescence [5-10]. Our biophysically detailed Purkinje neuron model of [11] can replicate this activity. It has a faithfully reconstructed morphology and it is computationally intensive. It has 1089 compartments and requires ~8 minutes of CPU time for 1 second of simulation (Intel core PC, i5). So, whilst it is appropriate for studying properties of the individual Purkinje cell, it is unfavorable for incorporation into cerebellar network simulations. In this paper, we introduce and employ a novel mathematical transform to produce a simpler, surrogate version of this model that has the same electrical properties, but a much lower computational overhead. It has just 2 compartments – a somatic compartment and a dendrite compartment. It runs >400 times faster than the full model. We demonstrate its expediency by showing that it can replicate the full model’s simulated response to alcohol (ethanol). Both models are available to researchers in the ModelDB [12; RRID: nif-0000-00004].

Seo and Suh [13] studied, *in vitro*, alcohol’s effect upon the firing pattern of Purkinje neurons in rat cerebellar slices. Prior to the application of alcohol some Purkinje cells were quiescent and others fired spikes spontaneously - some continuously and others in an oscillatory pattern of firing and quiescence. The application of alcohol shifted some quiescent cells into continuous spiking, it shifted some simple and oscillatory firing cells into quiescence and it shifted some oscillatory cells into simple, continuous spiking. These results are hard to reconcile. Seo and Suh [13] observe and describe this behaviour; they do not provide an explanation for it. Here, we use our Purkinje neuron model – with its detailed and reduced versions - to deliver a hypothesis that can accommodate these observations. We suggest that these results can be understood if alcohol action upon Purkinje cells is via an inhibition of its Na^+^/K^+^ pumps. There is some precedent to this as alcohol has been reported to inhibit Na^+^/K^+^ pumping in a number of other contexts [14-19].

The Na^+^/K^+^ pump uses the energy of one ATP molecule to exchange three intracellular Na^+^ ions for two extracellular K^+^ ions [20]. Thus the pump is electrogenic, extruding one net charge per cycle to hyperpolarize the membrane potential. We hypothesise, as we did in [11], that quiescent Purkinje cells are silent because the hyperpolarising action of the Na^+^/K^+^ pump clamps their intrinsic excitability and prevents spontaneous spike generation. Furthermore, in this manuscript we suggest that alcohol application inhibits the Na^+^/K^+^ pump, reduces its clamping action and permits spontaneous spiking. So, we suggest that spontaneously firing Purkinje cells are distinct from intrinsically quiescent Purkinje cells because of a lower Na^+^/K^+^ activity.

The Na^+^/Ca^2+^ exchanger current is net depolarizing (-1), inwardly passing 3 singly positive Na^+^ ions (3*[+1]) for the extrusion of every doubly positive Ca^2+^ ion (1*[+2]) [21]. The Na^+^/K^+^ pump current, by contrast, is net hyperpolarizing (+1). Our model incorporates our hypothesis (which is partially founded in the experimental work of Genet and Kado [22]) that, in Purkinje neurons, the hyperpolarizing Na^+^/K^+^ pump current electrically balances a depolarizing Na^+^/Ca^2+^ exchanger current. We suggest that alcohol inhibits the Na^+^/K^+^ pump and that this results in membrane depolarisation, because there is no longer this counterbalance to the depolarising Na^+^/Ca^2+^ exchanger current. This depolarisation can cause quiescence through a depolarisation block.

We suggest that the quiescent periods in oscillatory patterns of spontaneous Purkinje cell firing are produced by the electrogenic action of Na^+^/K^+^ pumping. This hypothesis is incorporated in the model. Relevantly, in rat cerebellar slices, an ouabain block of Na^+^/K^+^ pumps eradicates the quiescent mode in the trimodal and bimodal patterns of Purkinje cell activity, which might suggest that Na^+^/K^+^ pumping is their generative mechanism [11]. So, we suggest that alcohol inhibits the Na^+^/K^+^ pump and that this inhibition can switch a Purkinje cell from an oscillatory to a continuous firing mode. This is because there is no longer the requisite Na^+^/K^+^ pump activity to generate the quiescent periods.

This work aligns with our previous research [11,23-27] and suggests that the Na^+^/K^+^ pump controls the intrinsic activity mode of cerebellar Purkinje neurons. We previously modelled the Purkinje neuron in the context of its inputs and showed how the Na^+^/K^+^ pump may be directly involved in cerebellar information processing [27]. The cerebellum is responsible for motor co-ordination [28] and alcohol consumption corrupts motor control. We speculate that ingested alcohol disrupts cerebellar computation, and motor control, by inhibiting the Na^+^/K^+^ pump in cerebellar Purkinje neurons.

Our reduced Purkinje neuron model is useful for inclusion in network models. This is its first application. The second is that it can be used to advance the future development of the full, detailed Purkinje neuron model. In single neuron modelling, most parameters can be established by experimental recordings. However, there is a problematic exception. Maximal conductance (g_max_) values for ion currents are not well defined by present experimental techniques. In model construction, these are typically set as free parameters and tuned - often manually [29] (e.g. [30]) - by iteratively running the model with different values and observing which combination of these give the best fit between real and model cell output. Typically, a great number of model runs is required and with the computational expense of a detailed, morphologically faithful neuron model: each run takes a significant period of time. In the case of manual tuning, which can be the only route to an optimised model in some cases, it can be a very frustrating, time consuming and laborious process. Model tuning of two compartment models, with their faster run time, is an easier task. And with our two compartment Purkinje cell model, with its relation and concordance to the full Purkinje cell model: tuning gains in the two compartment model translates to equivalent gains in the full Purkinje cell model. Hence, the two compartment model can be used profitably as a proxy for tuning the full model. So, in this manuscript we present a reduced Purkinje neuron model. However, we anticipate that its principal legacy may be to facilitate the further development of more detailed, morphologically realistic Purkinje neuron models. We demonstrate this utility by using our reduced model to find parameter values that will permit our full model to replicate new behaviour; that observed experimentally for cerebellar Purkinje neurons in mice that have a BK channel genetic knockout [31,32]. These mice display cerebellar ataxia.

## Methods

Numerical simulations were performed with the NEURON 7.3 simulator [33; RRID: nif-0000-00081], using its backward Euler integration method and 25 μs time steps. Fixed time step integration was used throughout. Preliminary, feasibility testing with a variable time step method (CVODE in NEURON, [33]), even with a high error tolerance, did not produce significant speeding of simulations (data not shown).

### Reduction algorithm

Our reduction algorithm is a simple extension to an existing reduction algorithm [34,35]. Like the original formulation, it collapses the elaborate dendritic arbour into a smaller number of compartments whilst conserving axial resistance (*Ra* ). However, unlike prior formulations it collapses the dendritic tree to just a single compartment. Furthermore, it has an additional step whereby the length of the dendrite compartment is increased, with a compensatory change in radius to keep the compartment’s volume constant. This added step is required in order for the reduced Purkinje neuron model to replicate the behavior of the full Purkinje neuron model and express the trimodal activity pattern. The optimal dendrite length is found by manual tuning.

Recently an alternative extension to the algorithm of [34,35] has been published, in which the collapsing function incorporates a Strahler analysis of the dendritic tree [36,37]. However, this method typically produces reduced models of the cerebellar Purkinje cell that have tens of compartments and run, on average, ~20 times faster than the full model [37]. Our reduction method produces a faithful 2 compartment model that runs >400 times faster.

Our algorithm merges successive dendritic branches into an equivalent cylinder, preserving the axial resistance of the original branches. The radius (*R*) of the equivalent cylinder is given by:

1 $$ R\kern0.5em =\kern0.5em \sqrt{\underset{i}{\Sigma \kern0.5em {r}_i^2}} $$

where *r*_*i*_ are the radii of the collapsed branches for the equivalent cylinder. The length (*l*) of the equivalent cylinder is taken as an average of the lengths of the collapsed branches (*l*_i_) for that cylinder, weighted by their respective radii (*r*_i_):

2 $$ l\kern0.5em =\kern0.5em \frac{\underset{i}{\Sigma}{l}_i{r}_i}{\underset{i}{\Sigma}{r}_i} $$

The volume of the cylinder (*V*) is:

3 $$ V\kern0.5em =\kern0.5em \pi \kern0.5em \cdot \kern0.5em {R}^2\kern0.5em \cdot \kern0.5em l $$

We can then change the length of the cylinder (*l*) to a desired value and can compensate for this change, to keep the cylinder volume (*V*) constant, by adjusting the radius (*R*) value accordingly:

4 $$ R\kern0.5em =\kern0.5em \sqrt{\frac{V}{\pi \kern0.5em \cdot \kern0.5em l}} $$

This final manipulation is to confront the fact that the collapsing function does not conserve dendritic length. It gives the equivalent cylinder a short Length of 120.9 μm (and Diameter of 6.74 μm). This is a problem because Purkinje cell functioning requires a degree of uncoupling (distance) between somatic and dendritic events. With this length of dendritic compartment, the model cannot replicate the Purkinje cell’s trimodal firing pattern (data not shown). To address this, we set the equivalent cylinder with a Length of 529.29 μm and adjusted its Radius, to 1.61 μm, in order to keep the volume constant (Eq. 4). This optimal length was found by manual tuning and was with a specific axial resistivity (*Ra*) of 35.4 *Ωcm* (the default NEURON setting). If *Ra* is different, then the optimal dendrite compartment dimensions are different. For example, with a *Ra* value of 250 *Ωcm*, the dimensions are instead: Length = 240 μm; Radius = 4.78 μm.

We have previously published a 41 compartment and a 5 compartment model of the cerebellar Purkinje neuron [11]. These models can replicate the Purkinje cell’s trimodal pattern of firing. However, the model of this current manuscript is superior to these because a model with just 2 compartments is the more elegant solution; not least because it runs faster. In addition, we describe the 2 compartment model here in immense detail to maximize its utility to other researchers. This level of detail is missing in [11].

With our collapsing method, the membrane surface area of the reduced model is less than the original because, although axial resistance is conserved, surface area (and hence the membrane resistance and capacitance) is not. This is compensated for by introducing in the equivalent cylinder, a dendritic correction factor (*C*_d_), which rescales the current/pump/exchanger density values (*g*_i_) and membrane capacitance (*C*_m_) in the dendrites such that:

5 $$ {g}_i^{\hbox{'}}\kern0.5em =\kern0.5em {C}_d{g}_i $$

6 $$ {C}_i^{\hbox{'}}\kern0.5em =\kern0.5em {C}_d{C}_m $$

The dendritic correction factor *C*_d_ is the ratio of the total surface area of the dendritic segments to their equivalent cylinder (42,310/6,874 = 6.16). The model’s somatic compartment is not introduced into the collapsing algorithm and so in the reduced model it has the same dimensions as in the full model, and the *C*_d_ correction factor is not applied to any of its parameters.

Following the methodology of [35], our 2 compartment Purkinje cell model has *C*_d_ applied to the model parameter, *depth*, which sets the depth of the sub-membrane shell that Ca^2+^ diffuses in within the model dendrite.

7 $$ dept{h}^{\hbox{'}}\kern0.5em =\kern0.5em {C}_d depth $$

The *Q* parameter is a setting in the model’s description of extracellular K^+^ dynamics and in the full, 1089 compartment model it is 0.06. This parameter isn’t set or constrained by any feature of the reduction algorithm used and is not constrained by any experimental literature. It is a free parameter. We manually tuned this parameter to confer a best fit between reduced and full model output, ultimately assigning it a value of 0.0119. This is distinct from the value of 0.37 obtained by a *C*_d_ manipulation (0.06 * *C*_d_ = 0.37, where *C*_d_ is 6.16) but this is of no consequence, as there is no directive in the literature for applying *C*_d_ to such a parameter. If a different value of Ra is assigned, different dendrite dimensions are to be used (as aforementioned), and the Q parameter needs to be re-tuned. For example, if Ra = 250 *Ωcm*, *Q* = 0.0103 gives a best fit.

In the 1089 compartment model of Forrest et al. [11], spines were accounted for (implicitly) by setting the 1003 spiny dendrite compartments with a higher specific membrane capacitance (*C*_m_) value than the 84 smooth dendrite compartments (*C*_m_ = 0.8 μF/cm^2^ for the smooth dendrites, *C*_m_ = 1.5 μF/cm^2^ for the spiny dendrites). Our 2 compartment model makes no such account of spines; for its dendritic compartment: *C*_m_ = 0.8 μF/cm^2^ (before the *C*_d_ manipulation).

So, to summarize the reduction methodology: the full morphology is collapsed into just a soma compartment and a single dendrite compartment. This is done using the algorithm/NEURON code of [35], but modified to collapse the dendrites to just one compartment instead of three (this modified code is included with our model entry in ModelDB [12]). The length of this single dendrite compartment is then manually tuned to confer the best fit between reduced and full model output. For an inputted change in dendrite length, coding is used to automatically update other linked variables such that the user only has to tune this single parameter. For a change in dendrite length, the dendrite radius is automatically, accordingly updated to keep the dendrite volume constant. Furthermore, the surface area of the updated dendrite compartment is measured and a new, appropriate *C*_d_ value is automatically calculated and applied. This coding form is in the NEURON code of the model, available in ModelDB [12]. So, to re-iterate, only a single variable needs be adjusted to tune the reduced model; such that it can best reproduce the behaviour of the full model. We adjusted it manually but with it being just a single parameter, it is likely tractable to automatic tuning methods. Thus described is the principal, crucial step to tuning. However, as aforementioned, after the optimal dendrite length has been found, an even closer fit between reduced and model output can then be reached by going on to tune the Q model parameter. This latter step is specific to this cerebellar Purkinje neuron model but we anticipate that the prior steps are more general and could be used to produce 2 compartment models for other neuron types.

### Model equations

*C*_*m*_ is the membrane capacitance (0.8 μF/cm^2^), *I* is the current, *V* is the membrane potential in mV, *t* is time, *T* is temperature (36^*°*^*C*), *Ra* is the specific axial resistivity (35.4 *Ωcm* and g_max_ is the maximal conductance (“current density”).

The model currents have equations and kinetic parameters as described in their source literature with the exception of g_max_ values, which have been modified. g_max_ values, for the different currents, are shown in Table 1. These values are drawn from the detailed Purkinje model of [11] and were found by manual tuning. Below, as we describe each current, we also state the publication that it is drawn from.

**Table 1:** **Maximal current conductances (g**_**max**_ **; mS/cm** ^**2**^ **) for the Full, 1089 Compartment model and the Simplified, Two Compartment model**

**Current**	**Soma**	**Dendrite(1089 compartment model)**	**Dendrite (two compartment model)**
Resurgent Na^+^	156	0	0
P-type Ca^2+^	0.52	1.6	9.856
T-type Ca^2+^	0	0.6	3.696
E-type Ca^2+^	0	3.2	19.712
A-type K^+^	0	32	197.12
D-type K^+^	0	36	221.76
M-type K^+^	0	0.004	0.0246
Delayed rectifier K^+^	0	0.24	1.478
Bk K^+^	72.8	60	369.6
SK K^+^	10	0	0
K2 K^+^	0	0.16	0.986
Kv1.2 K^+^	0	1	6.16
Highly TEA sensitive K^+^	41.6	0	0
Moderately TEA sensitive K^+^	20.8	0	0
TEA insenstitive K^+^	41.6	0	0
hyperpolarization activated cation, I_h_	1.04	0.29	1.786
Leak	0.1	0.000079	0.000487

These models have the same g_max_ values at the soma but not in the dendrite. In the dendrite of the Two Compartment model, g_max_ values are different because they are multiplied by the dendritic correction factor, *C*_d_ (=6.16; *refer Methods*).

*m*, *h* and *z* are Hodgkin-Huxley “particles”/gates [38]; for example, for the *m* Hodgkin-Huxley gate:

8 $$ \frac{dm}{dt}=\frac{m_{\infty }-m}{\tau_m} $$

The voltage (and/or intracellular calcium) dependence of a Hodgkin-Huxley (H-H) current [38] can be expressed by stating, for each H-H gate (e.g. for the *m* gate), either [*m*_∞_, *m*_∞_] OR [*α*_*m*_, *β*_*m*_]. These entities are voltage (and/or intracellular calcium) dependent. The latter set can give the former set through the relations:

9 $$ {m}_{\infty }={\alpha}_m/\left({\alpha}_m+{\beta}_m\right) $$

10 $$ {\tau}_m=1/\left({\alpha}_m+{\beta}_m\right) $$

### Soma

The soma is a cylinder (length = 22 μm, diameter = 22 μm). E_K_ is the reversal potential for K^+^ (initiated at -88 mV), E_Na_ is the reversal potential for Na^+^ (initiated at +70 mV), E_Ca_ is the reversal potential for Ca^2+^ (initiated by the NEURON default value; +132 mV), E_L_ is the reversal potential for the Leak current (-70 mV), E_h_ is the reversal potential for the hyperpolarisation activated cation current (-30 mV), Intracellular Ca^2+^ concentration is initiated at the NEURON default of 5*10^−5^ mM; Extracellular Ca^2+^ concentration is initiated at the NEURON default of 2 mM. The somatic membrane voltage (*V*) is initiated at the NEURON default of -65 mV.

The soma has highly TEA sensitive (I_K_fast_), moderately TEA sensitive (I_K_mid_) and TEA insensitive (I_K_slow_) voltage-gated K^+^ currents, a BK voltage-and-Ca^2+^-gated K^+^ current (I_BK_), a resurgent Na^+^ current (I_Na-R_), a P-type Ca^2+^ current (I_CaP_), a hyperpolarization activated cation current (I_H_), a leak current (I_L_), a SK Ca^2+^-gated K^+^ current (I_SK_) and an intracellular Ca^2+^ dynamics abstraction. The soma has two Na^+^/K^+^ pump descriptions (I^s^_pump_ and i^s^_pump_) and a Na^+^/Ca^2+^ exchanger mechanism I^s^_ex_.

11 $$ {C}_m\cdot \frac{dV}{dt}=-\left({I}_{K\_ fast}+{I}_{K\_mid}+{I}_{K\_ slow}+{I}_{BK}+{I}_{CaP}+{I}_H+{I}_L+{I}_{NaR}+{I}_{SK}+{i}_{pump}^s+{I}_{pump}^s+{I}_{ex}^s+{I}_{transfer\_DS}\right) $$

#### Dendrite – Soma electrotonic current [39]

12 $$ {I}_{transfer\_DS}=\frac{\left({V}_D-{V}_S\right)}{R_{DS}} $$

13 $$ {R}_{DS}=\frac{Ra\cdot \left({L}_S/2\right)}{\pi \cdot {r_S}^2}+\frac{Ra\cdot \left({L}_D/2\right)}{\pi \cdot {r_D}^2} $$

V_D_ is the membrane voltage at the centre of the dendrite compartment, V_S_ is the membrane voltage at the centre of the soma compartment and R_DS_ is the axial Resistance between the two. Ra is the specific axial Resistivity, L_S_ and r_S_ are the length and radius of the soma respectively; L_D_ and r_D_ are the length and radius of the dendrite respectively.

#### Highly TEA sensitive K^+^ current [40]

14 $$ {I}_{K\_ fast}={g}_{\max}\cdot {m}^3\cdot h\cdot \left(V-{E}_K\right) $$

15 $$ {m}_{\infty }=\frac{1}{ \exp \left(-\frac{V--24}{15.4}\right)} $$

16 $$ {\tau}_m=\left\{\begin{array}{l}0.000103+0.0149\ast \exp \left(0.035\ast V\right)......................\left[V<-35 mV\right]\\ {}0.000129+1/\left[ \exp \left(\frac{V+100.7}{12.9}\right)+ \exp \left(\frac{V-56}{-23.1}\right)\right]......\left[V\ge -35 mV\right]\end{array}\right. $$

17 $$ {h}_{\infty }=0.31+\frac{1-0.31}{ \exp \left(-\frac{V--5.8}{-11.2}\right)} $$

18 $$ {\tau}_h=\left\{\begin{array}{l}1.22*{10}^{-5}+0.012\ast \exp \left[-{\left(\frac{V+56.3}{49.6}\right)}^2\right]......................\left[V\le 0 mV\right]\\ {}0.0012+0.0023* \exp \left(-0.141*V\right)..............................\left[V>0 mV\right]\end{array}\right. $$

#### Moderately TEA sensitive K^+^ current [40]

19 $$ {I}_{K\_mid}={g}_{\max}\cdot {m}^4\cdot \left(V-{E}_K\right) $$

20 $$ {m}_{\infty }=\frac{1}{ \exp \left(-\frac{V--24}{20.4}\right)} $$

21 $$ {\tau}_m=\left\{\begin{array}{l}0.000688+1/\left[ \exp \left(\frac{V+64.2}{6.5}\right)+ \exp \left(\frac{V-141.5}{-34.8}\right)\right]..............\left[V<-20 mV\right]\\ {}0.00016+0.0008* \exp \left(-0.0267*V\right)..............................\left[V\ge -20 mV\right]\end{array}\right. $$

#### TEA insensitive K^+^ current [40]

22 $$ {I}_{K\_ slow}={g}_{\max}\cdot {m}^4\cdot \left(V-{E}_K\right) $$

23 $$ {m}_{\infty }=\frac{1}{ \exp \left(-\frac{V--16.5}{18.4}\right)} $$

24 $$ {\tau}_m=0.000796+1/\left[ \exp \left(\frac{V+73.2}{11.7}\right)+ \exp \left(\frac{V-306.7}{-74.2}\right)\right] $$

#### P-type Ca^2+^ current [40]

25 $$ {I}_{CaP}={g}_{\max }*m*ghk $$

Goldman-Hodgkin-Katz (ghk) equation:

26 $$ ghk=\left(4*{P}_{C{a}^{2+}}\right)*\frac{V\cdot {F}^2}{R\cdot T}*\frac{{\left[C{a}^{2+}\right]}_i-{\left[C{a}^{2+}\right]}_o* \exp \left(\frac{-2\cdot F\cdot V}{R\cdot T}\right)}{1- \exp \left(\frac{-2\cdot F\cdot V}{R\cdot T}\right)} $$

P_Ca_^2+^ is 5*10^−5^cm/sec, [Ca^2+^]_i_ = 100 nM, [Ca^2+^]_o_ = 2 mM, T = 295 K, F is the Faraday constant and R is the gas constant. [Ca^2+^]_i_ and [Ca^2+^]_o_ are fixed constants, as seen by this equation – it does *not* access the changing value of [Ca^2+^]_i_ as set by the intracellular Ca^2+^ equations (given later).

27 $$ {m}_{\infty }=\frac{1}{ \exp \left(-\frac{V--19}{5.5}\right)} $$

28 $$ {\tau}_m=\left\{\begin{array}{l}0.000264+0.128* \exp \left(0.103*V\right).....................\left[V\le -50 mV\right]\\ {}0.000191+0.00376* \exp \left[-{\left(\frac{V+11.9}{27.8}\right)}^2\right]...........\left[V>-50 mV\right]\end{array}\right. $$

#### Hyperpolarisation activated cation current [40]

29 $$ {I}_H={g}_{\max}\cdot m\cdot \left(V-{E}_h\right) $$

30 $$ {m}_{\infty }=\frac{1}{ \exp \left(-\frac{V--90.1}{-9.9}\right)} $$

31 $$ {\tau}_m=0.19+0.72* \exp \left[-{\left(\frac{V+81.5}{11.9}\right)}^2\right] $$

#### BK type K^+^ current [40]

32 $$ {I}_{BK}={g}_{\max}\cdot {m}^3\cdot {z}^2\cdot h\cdot \left(V-{E}_K\right) $$

33 $$ {m}_{\infty }=\frac{1}{ \exp \left(-\frac{V--28.9}{6.2}\right)} $$

34 $$ {h}_{\infty }=0.085+\frac{1-0.085}{ \exp \left(-\frac{V--32}{-5.8}\right)} $$

35 $$ {\tau}_m=0.000505+1/\left[ \exp \left(\frac{V+86.4}{10.1}\right)+ \exp \left(\frac{V-33.3}{-10}\right)\right] $$

36 $$ {\tau}_h=0.0019+1/\left[ \exp \left(\frac{V+48.5}{5.2}\right)+ \exp \left(\frac{V-54.2}{-12.9}\right)\right] $$

37 $$ {z}_{\infty }=\frac{1}{1+\frac{0.001}{\left[C{a}^{2+}\right]}} $$

38 $$ {\tau}_z=1 $$

#### Leak current [40]

39 $$ {I}_L={g}_{\max }*\left(V-{E}_L\right) $$

#### Intracellular Ca^2+^ concentration [40]

[Ca^2+^] is calculated for the intracellular space within 100 nm of the membrane. [Ca^2+^] changes as I_Ca_^2+^ (negative by convention; inward currents are negative) brings Ca^2+^ into this space and as Ca^2+^ leaves by diffusion to the bulk cytoplasm. The diffusion rate constant,*β*, is set to 1/msec.

40 $$ \frac{d\left[C{a}^{2+}\right]}{dt}=\beta *\left[C{a}^{2+}\right] $$

[Ca^2+^] at time step, *t*:

41 $$ {\left[C{a}^{2+}\right]}_t={\left[C{a}^{2+}\right]}_{t-1}+\Delta t*\left(\frac{-(100)*{I}_{C{a}^{2+}}}{\left(2\cdot F\right)*\left( depth\cdot Area\right)}-\beta *{\left[C{a}^{2+}\right]}_{t-1}\right) $$

*F* is the Faraday constant, *depth* = 0.1 *μm* and membrane surface *Area* = 1,521*μm*^2^. [Ca^2+^] was constrained to not fall below 100 nM by coding of the form:

42 $$ if\left(\left[C{a}^{2+}\right]<100\right)\left\{\left[C{a}^{2+}\right]=100\right\} $$

#### Resurgent Na^+^ current [40,41]

43 $$ {I}_{NaR}=\kern0.5em {g}_{\max}\kern0.5em *\kern0.5em O\kern0.5em *\kern0.5em \left(V\kern0.5em \hbox{-} \kern0.5em {E}_{Na}\right)\ O\kern0.5em \mathrm{is}\ \mathrm{the}\ \mathrm{occupancy}\ \mathrm{of}\ \mathrm{the}\ \mathrm{Open}\ \mathrm{state}. $$

**Figure 1:**
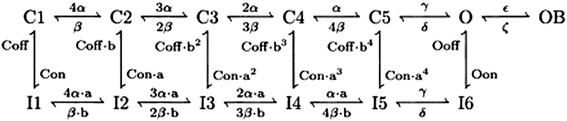
The Resurgent Na^+^ current is described by a Markov scheme [40,41]. [C1 to C5] denote sequential Closed states; O denotes the Open state. [I1 to I6] denote Inactivated states. OB denotes the state entered by a second mechanism of inactivation, which is hypothesized to be equivalent to Open Channel Block. The rate constants between states are given in Eq. [44], Eq. [45], Eq. [46], Eq. [47], Eq. [48] and Eq. [49].

This current is described by a Markov scheme, shown in Figure 1. The rate constants, labelled in Figure 1, are (ms^−1^):

44 $$ \alpha =150* \exp \left(\frac{V}{20}\right) $$

45 $$ \beta =3* \exp \left(\frac{2\cdot V}{20}\right) $$

$$ \gamma =150;\kern0.5em \delta =40;\kern0.5em  Con=0.005;\kern0.5em  Coff=0.5;\kern0.5em Oon=0.75;\kern0.5em  Ooff=0.005 $$

46 $$ a={\left(\frac{Oon}{Con}\right)}^{1/4} $$

47 $$ b={\left(\frac{Ooff}{Coff}\right)}^{1/4} $$

48 $$ \varepsilon =1.75 $$

49 $$ \zeta =0.03* \exp \left(\frac{2\cdot V}{25}\right) $$

#### SK type K^+^ current [42]

50 $$ {I}_{SK}={g}_{\max }*z*\left(V-{E}_K\right) $$

51 $$ z=\frac{1}{1+{\left(0.00019/\left[C{a}^{2+}\right]\right)}^4} $$

#### Na^+^/K^+^ pump [11]

The somatic Na^+^/K^+^ pump (density = d^s^_pump_, 1 mA/cm^2^) transports 3 Na^+^ out (i^s^_pump_Na_) for every 2 K^+^ in (i^s^_pump_K_). It has a fixed voltage (*V*) dependency and an exponential relation to intracellular Na^+^ concentration ([Na^+^]_i_). The affinity constant for Na^+^, K_Na_ = 40 mM.

52 $$ \left\{\begin{array}{l}{\mathrm{i}}_{\mathrm{pump}}^{\mathrm{s}} = {\mathrm{d}}_{\mathrm{pump}}^{\mathrm{s}}\left(\mathrm{V} + 75\right)\ /\ \left[\left(\mathrm{V} + 80\right)\left(1+ \exp \left(\frac{{\mathrm{K}}_{\mathrm{Na}}\hbox{-} {\left[{\mathrm{Na}}^{+}\right]}_{\mathrm{i}}}{1}\right)\right)\right]\\ {}{\mathrm{i}}_{\mathrm{pump}\_\mathrm{N}\mathrm{a}}^{\mathrm{s}} = 3{\mathrm{i}}_{\mathrm{pump}}^{\mathrm{s}}\\ {}{\mathrm{i}}_{\mathrm{pump}\_\mathrm{K}}^{\mathrm{s}} = \hbox{-} 2\ {\mathrm{i}}_{\mathrm{pump}}^{\mathrm{s}}\end{array}\right. $$

#### Na^+^/Ca^2+^ exchanger current and an electrically counterbalancing Na^+^/K^+^ pump current [11]

The soma has a simple Na^+^/Ca^2+^ exchanger mechanism (Eq. 53) and a simple Na^+^/K^+^ pump mechanism (Eq. 54). The Na^+^/Ca^2+^ exchanger current (I^s^_ex_net_) is *net* depolarizing (-1), inwardly passing 3 singly positive Na^+^ ions (3*[+1]) for the extrusion of every doubly positive Ca^2+^ ion (1*[+2]). By contrast, the Na^+^/K^+^ pump current (I^s^_pump_net_) is *net* hyperpolarizing (+1) in its transport of 3 Na^+^ out (3*[+1]) for every 2 K^+^ in (2*[+1]). The exchanger density is g^*s*^_ex_; the pump density is g^*s*^_pump_.

53 $$ \left\{\begin{array}{l}{\mathrm{I}}_{\mathrm{ex}\_\mathrm{N}\mathrm{a}}^s=-3\cdot \left[+1\right]\cdot {g}_{ex}^s\\ {}{\mathrm{I}}_{\mathrm{ex}\_\mathrm{C}\mathrm{a}}^{\mathrm{s}}=1\cdot \left[+2\right]\cdot {g}_{ex}^s\\ {}{I}_{ex\_net}^s=\left({I}_{ex\_Ca}^s-{I}_{ex\_Na}^s\right)\Rightarrow {g}_{ex}^s\cdot \left[-1\right]\Rightarrow -{g}_{ex}^s\end{array}\right. $$

54 $$ \left\{\begin{array}{l}{\mathrm{I}}_{\mathrm{pump}\_\mathrm{N}\mathrm{a}}^s=3\cdot \left[+1\right]\cdot {g}_{pump}^s\\ {}{\mathrm{I}}_{\mathrm{pump}\_\mathrm{K}}^{\mathrm{s}}=-2\cdot \left[+1\right]\cdot {g}_{pump}^s\\ {}{I}_{pump\_net}^s=\left({I}_{pump\_Na}^s-{I}_{pump\_K}^s\right)\Rightarrow {g}_{pump}^s\cdot \left[+1\right]\Rightarrow +{g}_{pump}^s\end{array}\right. $$

The Na^+^/K^+^ pump current of Eq. 54 largely, but incompletely, counterbalances the Na^+^/Ca^2+^ exchanger current of Eq. 53 – there is a *slight* mismatch [g^*s*^_ex_ = 0.511 mA/cm^2^, g^*s*^_pump_ = 0.5 mA/cm^2^] (Eq. 55) which permits a small *net* influx of Na^+^ ions.

55 $$ \left[{\mathrm{g}}_{\mathrm{ex}}^{\mathrm{s}} = {\mathrm{g}}_{\mathrm{pump}}^{\mathrm{s}} + 0.011\right]\kern0.5em \Rightarrow \kern0.5em \left[\hbox{-} {\mathrm{I}}_{\mathrm{ex}\_\mathrm{n}\mathrm{e}\mathrm{t}}^{\mathrm{s}}\kern0.5em \approx \kern0.5em +{\mathrm{I}}_{\mathrm{pump}\_\mathrm{n}\mathrm{e}\mathrm{t}}^{\mathrm{s}}\right] $$

#### Intracellular Na^+^ concentration [11]

[Na^+^]_i_ is initiated at 10 mM and then changes in time,

56 $$ \frac{\partial {\left[N{a}^{+}\right]}_i}{\partial t}=\frac{I_{Na\_net}}{\left[d\cdot F\cdot (10000)\right]/4}+\frac{D{\partial}^2{\left[N{a}^{+}\right]}_i}{{\left(\partial x\right)}^2} $$

57 $$ {\left({I}_{Na\_net}\right)}_{\left[t\right]}={\left({I}_{Na\_ in}-{I}_{Na\_ out}\right)}_{\left[t-\tau \right]},\tau = 5s $$

58 $$ {I}_{Na\_ in}={I}_{Na-R}+{I}_{ex\_Na}^s $$

59 $$ {I}_{Na\_ out}\kern0.5em =\kern0.5em {i^s}_{pump\_Na}\kern0.5em +\kern0.5em {I_{pump}^s}_{\_Na} $$

*F* is the Faraday constant, *d* is the somatic diameter and *D* is the diffusivity constant (0.6 μm^2^/ms). The second term on the Right Hand Side (RHS) of Eq. 56 accounts for longitudinal diffusion of Na^+^ out of the soma compartment, along the longitudinal distance (*x*). The effects of this term are fairly negligible and it could be dropped to quicken simulation speeds. *I*_*Na_net*_ is the difference between Na^+^ current flowing into the soma *(I*_*Na_in*_*; through I*_*Na-R*_*and I*^*s*^_*ex_Na*_*)* and Na^+^ current pumped out of the soma by the Na^+^/K^+^ pump (*I*_*Na_out*_), lagged by parameter τ = 5 s. Intracellular Na^+^ stimulates the Na^+^/K^+^ pump and this lag *τ* accounts for the duration of sodium’s diffusion from channels to pumps. i^s^_pump_Na_ and I^s^_pump_Na_ are Na^+^ currents produced by the pumping action of the Na^+^/K^+^ pump at the soma, set by Eq. 52 and Eq. 54 respectively. “Catch coding” is applied:

60 $$ \mathrm{if}\ \left({\left[\mathrm{N}{\mathrm{a}}^{+}\right]}_{\mathrm{i}} < 10\ \right)\ \left[{\left[\mathrm{N}{\mathrm{a}}^{+}\right]}_{\mathrm{i}} = 10\ \right] $$

61 $$ \mathrm{if}\ \left({\mathrm{E}}_{\mathrm{Na}} < 70\ \right)\ \left[{\mathrm{E}}_{\mathrm{Na}} = 70\right] $$

### Dendrite

The dendrite is a cylinder (length = 529.29 μm, diameter = 3.22 μm). E_K_ is the reversal potential for K^+^ (initiated by the NEURON default value; -77 mV), E_Na_ is the reversal potential for Na^+^ (initiated by the NEURON default value; +50 mV), E_Ca_ is the reversal potential for Ca^2+^ (initiated by the NEURON default value; +132 mV), E_L_ is the reversal potential for the Leak current (-80 mV), E_h_ is the reversal potential for the hyperpolarisation activated cation current (-32.9 mV). Intracellular Ca^2+^ concentration is initiated at 4*10^−5^ mM; Extracellular Ca^2+^ concentration is initiated at 2.4 mM. Extracellular Na^+^ concentration is set by the NEURON default value; +140 mM.

All the mechanisms in the dendrite are distinct from those in the soma. The dendrite has hyperpolarization activated cation current (I_H_); T-type (I_CaT_), Class-E (I_CaE_) and P-type (I_CaP_) voltage-gated Ca^2+^ currents; a leak current (I_L_); A-type (I_KA_), D-type (I_KD_), M-type (I_KM_), Delayed Rectifier (I_DR_) and Kv1.2 (I_Kv1.2_) voltage-gated K^+^ currents; BK (I_BK_) and K2 (I_K2_) type voltage-and-Ca^2+^-gated K^+^ currents and an intracellular Ca^2+^ dynamics abstraction. The dendrite has two Na^+^/K^+^ pump descriptions (I^d^_pump_ and i^d^_pump_) and a Na^+^/Ca^2+^ exchanger mechanism I^d^_ex_.

62 $$ {C}_m\cdot \frac{dV}{dt}=-\left(\begin{array}{l}{I}_{CaT}+{I}_{CaE}+{I}_{CaP}+{I}_H+{I}_{Kv1.2}+{I}_{KA}+{I}_{KM}+{I}_{KD}+{I}_{DR}+{I}_{BK}+{I}_{K2}+{I}_L+{i}_{pump}^d\\ {}+{I}_{pump}^d+{I}_{ex}^d+{I}_{transfer\_SD}\end{array}\right) $$

#### Soma - dendrite electrotonic current [39]

63 $$ {I}_{transfer\_SD}=\frac{\left({V}_S-{V}_D\right)}{R_{SD}} $$

64 $$ {R}_{SD}=\frac{Ra\cdot \left({L}_S/2\right)}{\pi \cdot {r_S}^2}+\frac{Ra\cdot \left({L}_D/2\right)}{\pi \cdot {r_D}^2} $$

V_S_ is the membrane voltage at the centre of the soma compartment, V_D_ is the membrane voltage at the centre of the dendrite compartment and R_SD_ is the axial Resistance between the two. Ra is the specific axial Resistivity, L_S_ and r_S_ are the length and radius of the soma respectively; L_D_ and r_D_ are the length and radius of the dendrite respectively.

#### T-type Ca^2+^ current [43]

65 $$ {I}_{CaT}={g}_{\max}\cdot m\cdot h\cdot \left(V-{E}_{Ca}\right)\kern1.75em ;\kern1.25em {\mathrm{E}}_{\mathrm{Ca}}\mathrm{is}\ \mathrm{f}\mathrm{ixed}\ \mathrm{at} + 135\ \mathrm{mV}\ \mathrm{f}\mathrm{o}\mathrm{r}\ \mathrm{this}\ \mathrm{current}. $$

66 $$ {\alpha}_m=\frac{2.6}{1+ \exp \left(\frac{V+21}{-8}\right)} $$

67 $$ {\beta}_m=\frac{0.18}{1+ \exp \left(\frac{V+40}{4}\right)} $$

68 $$ {\alpha}_h=\frac{0.0025}{1+ \exp \left(\frac{V+40}{8}\right)} $$

69 $$ {\beta}_h=\frac{0.19}{1+ \exp \left(\frac{V+50}{-10}\right)} $$

$mt={3}^{\frac{T-37}{10}}$; *T* is temperature in degrees centigrade (36).

70 $$ {\tau}_m=\frac{1}{\left({\alpha}_m+{\beta}_m\right)\cdot mt} $$

71 $$ {\tau}_h=\frac{1}{\left({\alpha}_h+{\beta}_h\right)\cdot mt} $$

#### E-type Ca^2+^ current [43]

72 $$ {I}_{CaE}={g}_{\max}\cdot m\cdot h\cdot \left(V-{E}_{Ca}\right);{\mathrm{E}}_{\mathrm{Ca}}\mathrm{is}\ \mathrm{f}\mathrm{ixed}\ \mathrm{at} + 135\ \mathrm{mV}\ \mathrm{f}\mathrm{o}\mathrm{r}\ \mathrm{this}\ \mathrm{current}. $$

73 $$ {\alpha}_m=\frac{2.6}{1+ \exp \left[\left(V+7\right)/-8\right]} $$

74 $$ {\beta}_m=\frac{0.18}{1+ \exp \left[\left(V+26\right)/4\right]} $$

75 $$ {\alpha}_h=\frac{0.0025}{1+ \exp \left[\left(V+32\right)/8\right]} $$

76 $$ {\beta}_h=\frac{0.19}{1+ \exp \left[\left(V+42\right)/-10\right]} $$

$mt={3}^{\frac{T-37}{10}}$; *T* is temperature in degrees centigrade (36).

77 $$ {m}_{\exp }=1- \exp \left(\frac{\left[-dt*mt\right]\cdot \left[{\alpha}_m+{\beta}_m\right]}{4}\right) $$

78 $$ {h}_{\exp }=1- \exp \left(\frac{\left[-dt*mt\right]\cdot \left[{\alpha}_h+{\beta}_h\right]}{10}\right) $$

#### P-type Ca^2+^ current [43]

79 $$ {I}_{CaP}={g}_{\max}\cdot m\cdot \left(V-{E}_{Ca}\right);{\mathrm{E}}_{\mathrm{Ca}}\mathrm{is}\ \mathrm{f}\mathrm{ixed}\ \mathrm{at} + 135\ \mathrm{mV}\ \mathrm{f}\mathrm{o}\mathrm{r}\ \mathrm{this}\ \mathrm{current}. $$

80 $$ {\alpha}_m=\frac{8.5}{1+ \exp \left(\left[V+-8\right]/-12.5\right)} $$

81 $$ {\beta}_m=\frac{35}{1+ \exp \left(\left[V+74\right]/14.5\right)} $$

$mt={3}^{\frac{T-37}{10}}$; *T* is temperature in degrees centigrade (36).

82 $$ {\tau}_m=\frac{1}{\left({\alpha}_m+{\beta}_m\right)\cdot mt} $$

#### Hyperpolarisation activated cation current [44]

83 $$ {I}_h={g}_{\max}\cdot m\cdot \left(V-{E}_h\right);\ {\mathrm{E}}_{\mathrm{h}} = \hbox{-} 32.9\ \mathrm{mV} $$

84 $$ {\tau}_m=\frac{1}{ \exp \left(-17.9-0.116\cdot V\right)+ \exp \left(-1.84+0.09\cdot V\right)}+100 $$

85 $$ {m}_{\infty }=\frac{1}{1+ \exp \left[\left(V+84.1\right)/10.2\right]} $$

86 $$ {m}_{\exp }=1- \exp \left(\frac{-dt}{\tau_m}\right) $$

#### Kv1.2 K^+^ current [45]

87 $$ {I}_{Kv1.2}={g}_{\max}\cdot {m}^4\cdot \left(V-{E}_K\right) $$

88 $$ {\alpha}_m=0.12899* \exp \left(\frac{-\left(V+45\right)}{-33.90877}\right) $$

89 $$ {\beta}_m=0.12899* \exp \left(\frac{-\left(V+45\right)}{12.42101}\right) $$

$mt={3}^{\frac{T-22}{10}}$; *T* is temperature in degrees centigrade (36).

90 $$ {\tau}_m=\frac{1}{\left({\alpha}_m+{\beta}_m\right)\cdot mt} $$

#### A-type K^+^ current [43]

91 $$ {I}_{KA}={g}_{\max}\cdot {m}^4\cdot h\cdot \left(V-{E}_K\right) $$

92 $$ {\alpha}_m=\frac{1.4}{1+ \exp \left(\left[V+27\right]/-12\right)} $$

93 $$ {\beta}_m=\frac{0.49}{1+ \exp \left(\right[V+30/4\Big)} $$

94 $$ {\alpha}_h=\frac{0.00175}{1+ \exp \left(\right[V+50/8\Big)} $$

95 $$ {\beta}_h=\frac{0.49}{1+ \exp \left(\right[V+13/-10\Big)} $$

$mt={3}^{\frac{T-37}{10}}$; *T* is temperature in degrees centigrade (36).

96 $$ {\tau}_m=\frac{1}{\left({\alpha}_m+{\beta}_m\right)\cdot mt} $$

97 $$ {\tau}_h=\frac{1}{\left({\alpha}_h+{\beta}_h\right)\cdot mt} $$

#### M-type K^+^ current [43]

98 $$ {I}_{KM}={g}_{\max}\cdot m\cdot \left(V-{E}_K\right) $$

$ft={2.3}^{\frac{T-36}{10}}$; *T* is temperature in degrees centigrade (36).

99 $$ {\tau}_m=\frac{1000/ ft}{3.3\cdot \left({e}^{+\left(V+35\right)/40}+{e}^{-\left(V+35\right)/20}\right)} $$

100 $$ {m}_{\infty }=\frac{1}{1+{e}^{-\left(V+35\right)/10}} $$

#### D-type K^+^ current [43]

101 $$ {I}_{KD}={g}_{\max}\cdot m\cdot h\cdot \left(V-{E}_K\right) $$

102 $$ {\alpha}_m=\frac{8.5}{1+ \exp \left(\left[V+17\right]/-12.5\right)} $$

103 $$ {\beta}_m=\frac{35}{1+ \exp \left(\left[V+99\right]/14.5\right)} $$

104 $$ {\alpha}_h=\frac{0.0015}{1+ \exp \left(\left[V+89\right]/8\right)} $$

105 $$ {\beta}_h=\frac{0.0055}{1+ \exp \left(\left[V+83\right]/-8\right)} $$

106 $$ {m}_{\infty }={\alpha}_m/\left({\alpha}_m+{\beta}_m\right) $$

107 $$ {h}_{\infty }={\alpha}_h/\left({\alpha}_h+{\beta}_h\right) $$

$mt={3}^{\frac{T-37}{10}}$; *T* is temperature in degrees centigrade (36).

108 $$ {m}_{\exp }=1- \exp \left(\frac{\left[-dt*mt\right]\cdot \left[{\alpha}_m+{\beta}_m\right]}{10}\right) $$

109 $$ {h}_{\exp }=1- \exp \left(\left[-dt*mt\right]\cdot \left[{\alpha}_h+{\beta}_h\right]\cdot 1.6\right) $$

These equations are based upon the NEURON code of [43], as opposed to the equations in their associated journal paper; there is some minor discrepancy between the two. This code was sourced from ModelDB [12].

#### Delayed Rectifier type K^+^ current [43]

110 $$ {I}_{DR}={g}_{\max}\cdot {m}^4\cdot \left(V-{E}_K\right) $$

111 $$ {\alpha}_m=0.1\cdot vtrap $$

112 $$ catch= fabs\left(\frac{-\left(V+55\right)}{10}\right) $$

Where fabs(x) returns the absolute value of a floating point number; the absolute value of its argument (|x|).

113 $$ vtrap=\left\{\begin{array}{l}10\cdot \left(1-\frac{-\left(V+55\right)}{10}/2\right)....................\left[ catch<1{e}^{-6}\right]\\ {}\frac{-\left(V+55\right)}{ \exp \left(\frac{-\left(V+55\right)}{10}\right)-1}.......................\left[ catch\ge 1{e}^{-6}\right]\end{array}\right. $$

114 $$ {\beta}_m=0.125\cdot \exp \left(\frac{-\left(V+65\right)}{80}\right) $$

$mt={3}^{\frac{T-37}{10}}$; *T* is temperature in degrees centigrade (36).

115 $$ {m}_{\exp }=1- \exp \left(-dt\cdot mt\cdot \left[{\alpha}_m+{\beta}_m\right]\right) $$

#### BK type K^+^ current [43]

116 $$ {I}_{BK}={g}_{\max}\cdot m\cdot {z}^2\cdot \left(V-{E}_K\right) $$

117 $$ {\alpha}_m=7.5 $$

118 $$ {\beta}_m=\frac{0.11}{ \exp \left(\left[V+-35\right]/14.9\right)} $$

119 $$ {m}_{\exp }=1- \exp \left(-dt\cdot \left[{\alpha}_m+{\beta}_m\right]\right) $$

120 $$ {\alpha}_z=1 $$

121 $$ {\beta}_z=\frac{400}{\left[C{a}^{2+}\right]*1000} $$

122 $$ {\tau}_z=10 $$

123 $$ {z}_{\exp }=1- \exp \left(\frac{-dt}{\tau_z}\right) $$

These equations are based upon the NEURON code of [43], as opposed to the equations in their associated journal paper; there is some minor discrepancy between the two. This code was sourced from ModelDB [12].

#### K2 type K^+^ current [43]

124 $$ {I}_{K2}={g}_{\max}\cdot m\cdot {z}^2\cdot \left(V-{E}_K\right) $$

125 $$ {\alpha}_m=25 $$

126 $$ {\beta}_m=\frac{0.075}{ \exp \left(\left[V+5\right]/10\right)} $$

127 $$ {m}_{\exp }=1- \exp \left(-dt\cdot \left[{\alpha}_m+{\beta}_m\right]\right) $$

128 $$ {\alpha}_z=1 $$

129 $$ {\beta}_z=\frac{20}{\left[C{a}^{2+}\right]*1000} $$

130 $$ {\tau}_z=10 $$

132 $$ {z}_{\exp }=1- \exp \left(\frac{-dt}{\tau_z}\right) $$

These equations are based upon the NEURON code of [43], as opposed to the equations in their associated journal paper; there is some minor discrepancy between the two. This code was sourced from ModelDB [12].

#### Leak current [43]

133 $$ {I}_L=\kern0.5em {g}_{\max }*\kern0.5em \left(V-{E}_L\right);{\mathrm{E}}_{\mathrm{L}}\mathrm{is}\ \hbox{-} 80\ \mathrm{mV}\ \mathrm{f}\mathrm{o}\mathrm{r}\ \mathrm{this}\ \mathrm{current}\ \mathrm{in}\ \mathrm{the}\ \mathrm{dendrite}. $$

#### Intracellular Ca^2+^ dynamics [43]

134 $$ \frac{d{\left[C{a}^{2+}\right]}_i}{dt}\kern0.5em =\kern0.5em  chan\kern0.5em +\kern0.5em \left(\frac{-\kern0.5em kt\kern0.5em *\kern0.5em {\left[C{a}^{2+}\right]}_i}{{\left[C{a}^{2+}\right]}_i\kern0.5em +\kern0.5em kd}\right)\kern0.5em +\kern0.5em \left(\frac{y\kern0.5em -\kern0.5em {\left[C{a}^{2+}\right]}_i}{ta{u}_r}\right) $$

135 $$ chan=\left(\frac{-(10000)*{I}_{C{a}^{2+}}}{2*F* depth}\right) $$

136 $$ if\left( chan<0\right)\left\{ chan=0\right\} $$

where [Ca^2+^]_i_ is the intracellular Ca^2+^ concentration in a supra-membrane shell of *depth* = 0.1 μm, *F* is the Faraday constant, ${I}_{C{a}^{2+}}$ is the Ca^2+^ membrane current (negative by convention; inward currents are negative), *kt* = 4*10^−5^ mM/ms, *kd* = 4*10^−5^ mM, tau_r_ = 2 ms and y = 4*10^−5^ mM.

#### Na^+^/K^+^ pump [11]

The dendritic Na^+^/K^+^ pump (density = d^*d*^_pump_, 0.001 mA/cm^2^) has a 3Na^+^:2 K^+^ stoichiometry, no voltage dependency and a hyperbolic relation to extracellular K^+^ concentration ([K^+^]_o_.

137 $$ \left\{\begin{array}{l}{\mathrm{i}}_{\mathrm{pump}}^d = {\mathrm{d}}_{\mathrm{pump}}^d/\ \left(1+\left(2.245/\ {\left[{\mathrm{K}}^{+}\right]}_{\mathrm{o}}\right)\right)\\ {}{\mathrm{i}}_{\mathrm{pump}\_\mathrm{N}\mathrm{a}}^d = 3{\mathrm{i}}_{\mathrm{pump}}^d\\ {}{\mathrm{i}}_{\mathrm{pump}\_\mathrm{K}}^d = \hbox{-} 2\ {\mathrm{i}}_{\mathrm{pump}}^d\end{array}\right. $$

#### Na^+^/Ca^2+^ exchanger current and an electrically counterbalancing Na^+^/K^+^ pump current [11]

The dendrite has a simple Na^+^/Ca^2+^ exchanger mechanism (Eq. 138) and a simple Na^+^/K^+^ pump mechanism (Eq. 139). The Na^+^/Ca^2+^ exchanger current is *net* depolarizing (-1), inwardly passing 3 singly positive Na^+^ ions (3*[+1]) for the extrusion of every doubly positive Ca^2+^ ion (1*[+2]). By contrast, the Na^+^/K^+^ pump current is *net* hyperpolarizing (+1) in its transport of 3 Na^+^ out (3*[+1]) for every 2 K^+^ in (2*[+1]).

138 $$ \left\{\begin{array}{l}{\mathrm{i}}_{\mathrm{ex}\_\mathrm{N}\mathrm{a}}^d = \hbox{-} 3\cdot \kern0.5em \left[+1\right]\cdot {g}_{ex}^d\\ {}{\mathrm{i}}_{\mathrm{ex}\_\mathrm{C}\mathrm{a}}^{\mathrm{d}} = 1\cdot \left[+2\right]\cdot {g}_{ex}^d\\ {}{\mathrm{i}}_{ex\_Net}^{\mathrm{d}} = \left({\mathrm{i}}_{ex\_Ca}^{\mathrm{d}}\kern0.5em -\kern0.5em {\mathrm{i}}_{ex\_Na}^{\mathrm{d}}\right)\kern0.5em \Rightarrow \kern0.5em {g}_{ex}^d\kern0.5em \cdot \left[-1\right]\kern0.5em \Rightarrow \kern0.5em -\kern0.5em {g}_{ex}^d\end{array}\right. $$

139 $$ \left\{\begin{array}{l}{\mathrm{I}}_{\mathrm{pump}\_\mathrm{N}\mathrm{a}}^d = 3\cdot \kern0.5em \left[+1\right]\cdot {g}_{pump}^d\\ {}{\mathrm{I}}_{\mathrm{pump}\_\mathrm{K}}^{\mathrm{d}} = 2\cdot \left[+1\right]\cdot {g}_{pump}^d\\ {}{I}_{pump\_net}^d = \left({I}_{pump\_Na}^d\kern0.5em -\kern0.5em {I}_{pump\_K}^d\right)\kern0.5em \Rightarrow \kern0.5em {g}_{pump}^d\kern0.5em \cdot \left[+1\right]\kern0.5em \Rightarrow \kern0.5em -\kern0.5em {g}_{pump}^d\end{array}\right. $$

g^*d*^_ex_ and g^*d*^_pump_ are Na^+^/Ca^2+^ exchanger and Na^+^/K^+^ pump membrane current densities (respectively) in the dendrite and their equality at 0.0021 mA/cm^2^ ensures an electrical counterbalance So, the model dendrite has a Na^+^/Ca^2+^ exchanger current and an electrically counterbalancing Na^+^/K^+^ pump current:

140 $$ \left[-{g}_{\mathrm{ex}}^{\mathrm{d}}\kern0.5em =\kern0.5em +\kern0.5em {g}_{\mathrm{pump}}^{\mathrm{d}}\right]\kern0.5em \Rightarrow \kern0.5em \left[-{I}_{\mathrm{ex}\_\mathrm{n}\mathrm{e}\mathrm{t}}^{\mathrm{d}}\kern0.5em =\kern0.5em +\kern0.5em {I}_{\mathrm{pump}\_\mathrm{n}\mathrm{e}\mathrm{t}}^{\mathrm{d}}\right] $$

#### Extracellular K^+^ dynamics [11]

Extracellular K^+^ concentration ([K^+^]_o_) to the dendrite is initiated at 2 mM and then changes in time, *t*, according to the relationship:

141 $$ \frac{d{\left[{K}^{+}\right]}_0}{dt}=\frac{(1000)\cdot Q\cdot {I}_{K\_net}}{F\cdot wid} $$

142 $$ {I}_{K\_net}\kern0.5em =\kern0.5em {I}_{K\_ out}\kern0.5em -\kern0.5em {I}_{K\_ in} $$

143 $$ {I}_{K\_ out}={I}_{KD}+{I}_{KA}+{I}_{KM}+{I}_{DR}+{I}_{BK}+{I}_{K2}+{I}_{Kv1.2} $$

144 $$ {I}_{K\_ in}\kern0.5em =\kern0.5em {i}_{pump\_K}^d\kern0.5em +\kern0.5em {I}_{pump\_K}^d $$

where *F* is the Faraday constant, *wid* is the thickness of an extracellular region around the compartment that K^+^ accumulates in (70*10^−3^ μm), Q is a K^+^ accumulation factor (0.0119) and *I*_*K_net*_ (Eq. 142) is the difference between K^+^ current flowing out of the compartment (*I*_*K_out*_; through gated K^+^ conductances, Eq. 143) and K^+^ current pumped into the compartment (*I*_*K_in*_; by the Na^+^/K^+^ pump, Eq. 144). i^d^_pump_K_ is set by Eq. 137; I^d^_pump_K_ is set by Eq. 139. “Catch coding” is applied to [K^+^]_o_:

145 $$ \mathrm{if}\ \left({\left[{\mathrm{K}}^{+}\right]}_{\mathrm{o}}>3.03\right)\ \left[{\left[{\mathrm{K}}^{+}\right]}_{\mathrm{o}} = 3.03\right] $$

146 $$ \mathrm{if}\ \left({\left[{\mathrm{K}}^{+}\right]}_{\mathrm{o}}<2\right)\ \left[{\left[{\mathrm{K}}^{+}\right]}_{\mathrm{o}} = 2\right] $$

Eq. 141 is based upon the NEURON code of [11], as opposed to the relevant equation in their associated journal paper; there is a minor discrepancy between the two (the paper does not have the dimensionality factor (1000) in its equation).

## Results

**Figure 2:**
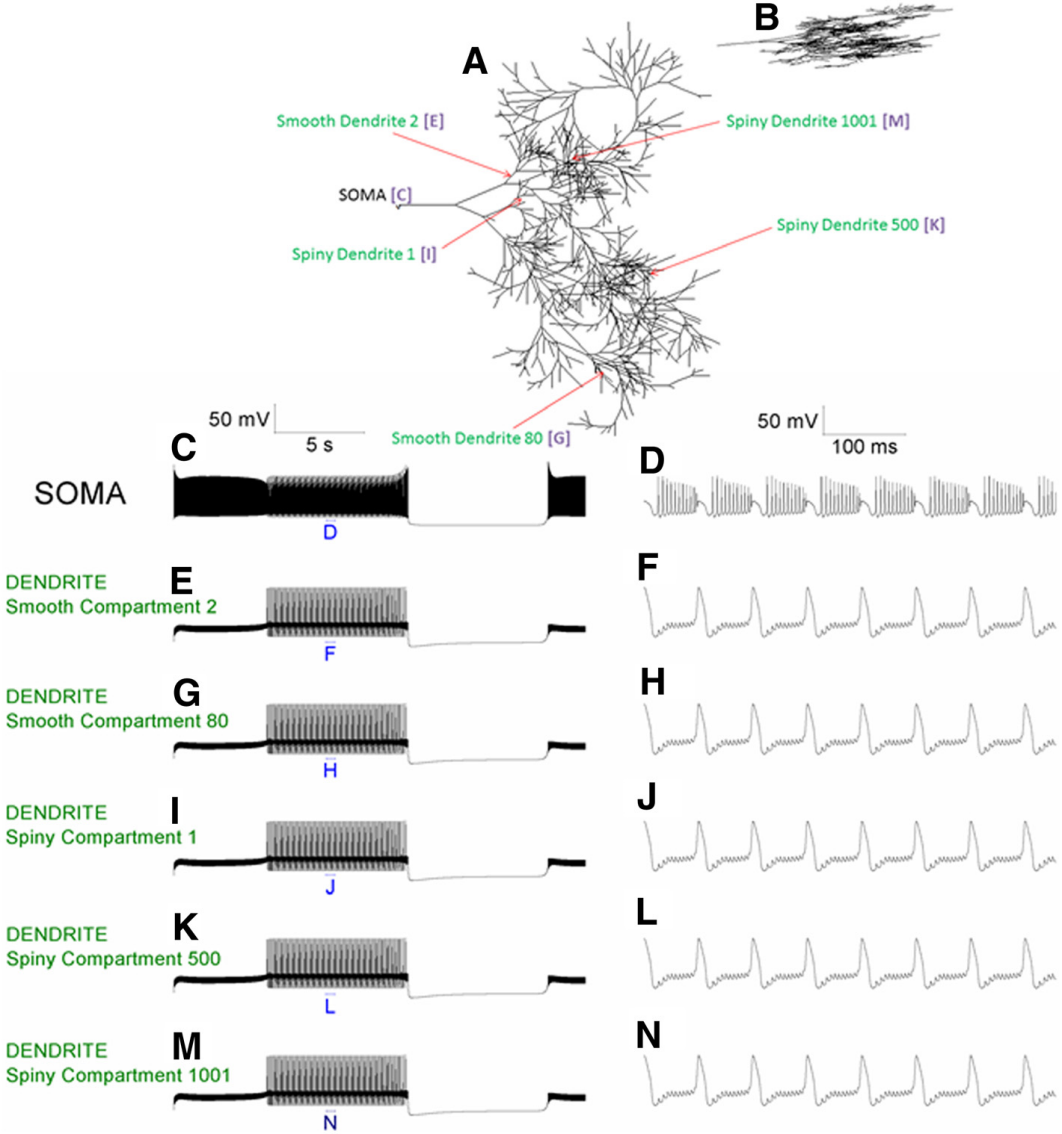
The 1089 compartment Purkinje neuron model can replicate the spontaneous trimodal firing pattern of real Purkinje neurons. ***A***, Morphology of the Purkinje neuron model. ***B***, Morphology of the model in profile, showing its planar character. ***C***, Spontaneous, single trimodal repeat recorded at the soma, with component Tonic, Burst and Quiescent modes discernible. ***D***, The labelled part of Panel **(C)** at higher resolution, showing a number of somatic bursts from the Burst firing mode. ***E***, The same period as shown in Panel **(C)** but recorded from a point in the dendritic tree: Smooth Compartment 2. Its dendritic location is presented in Panel **(A)**. ***F***, The labelled part of Panel **(E)** at higher resolution, showing a number of high-threshold dendritic Ca^2+^ spikes. ***G***, Recording from Smooth Compartment 80. ***H***, The labelled part of Panel **(G)** at higher resolution. ***I***, Recording from Spiny Compartment 1. ***J***, The labelled part of Panel **(I)** at higher resolution. ***K***, Recording from Spiny Compartment 500. ***L***, The labelled part of Panel **(K)** at higher resolution. ***M***, Recording from Spiny Compartment 1001. ***N***, The labelled part of Panel **(M)** at higher resolution. This Figure shows that all points in the dendritic tree have equivalent activity. **Panels C, E, G, I, K, M** are scaled by the first scale bar (50 mV, 5 s). **Panels D, F, H, J, L, N** are scaled by the second scale bar (50 mV, 100 ms).

The 1089 compartment model can replicate the Purkinje neuron’s trimodal pattern of spontaneous activity. The trimodal repeat motif consists of tonic spiking, bursting and quiescent phases (Figure 2). No stimulation protocol is required to observe this model activity; it is spontaneous. The model’s morphology is shown (2A) and its planar character can be observed as it is shown in profile (2B). This morphology is a reconstruction of that from a real Purkinje neuron [11]. The electrical activity at the soma is presented (2C) and the tonic, burst and quiescent modes can be identified. Somatic bursts are shown at a higher resolution in (2D). The electrical activities of a number of arbitrary points in the dendritic tree are presented; their locations are labelled in the morphology depiction of (2A). In the left column their activity is shown in entirety (panels: E, G, I, K, M), in the right column their dendritic high-threshold calcium spikes are shown at higher resolution (panels: F, H, J, L, N). One can note that these dendritic spikes align with, and cause, bursts at the soma. Figure 2 shows that all dendritic compartments in the 1089 compartment model have an equivalent electrical activity.

**Figure 3:**
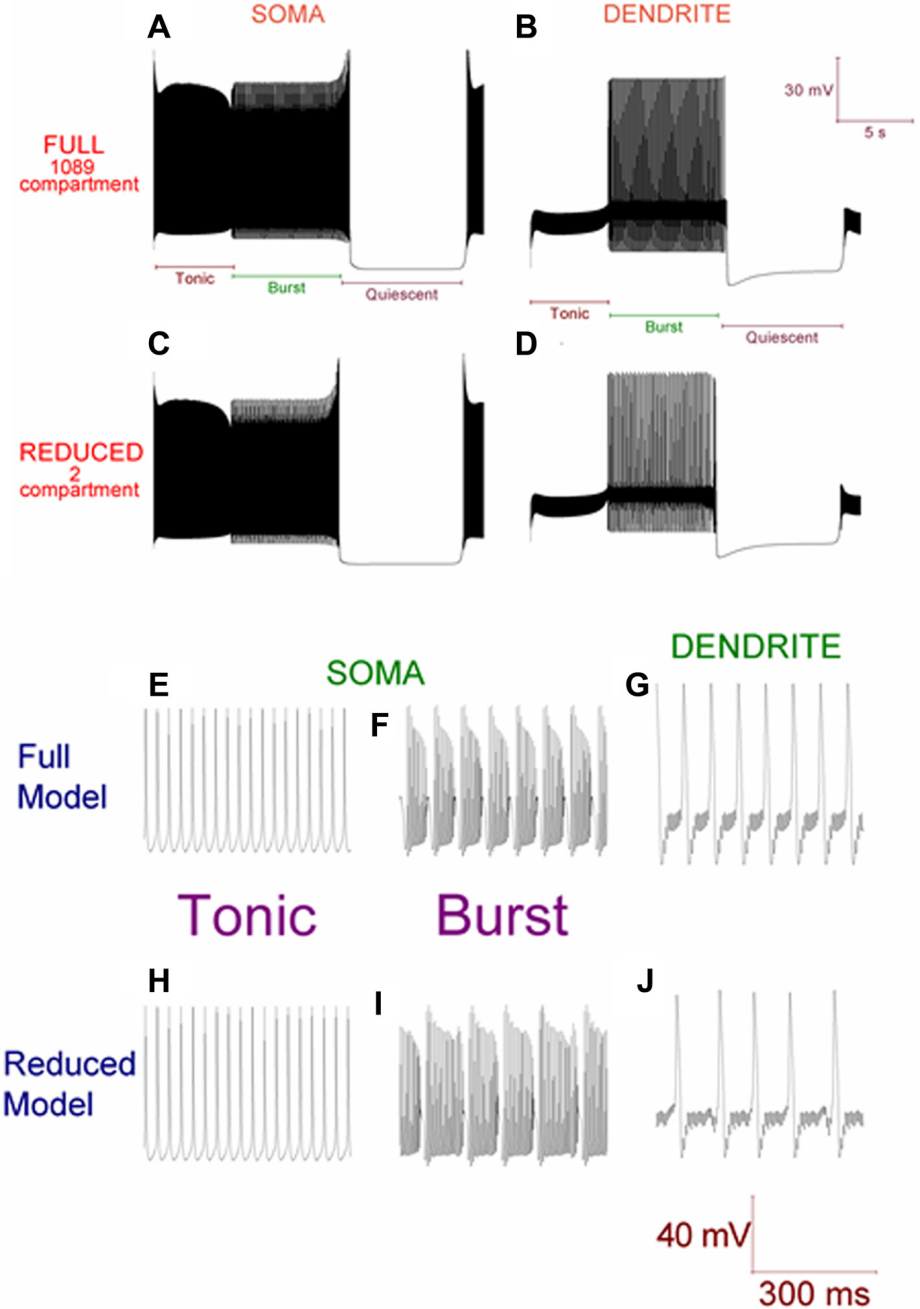
The 2 compartment Purkinje neuron model can replicate the spontaneous trimodal firing pattern of the 1089 compartment Purkinje neuron model. ***A***, Spontaneous, single trimodal repeat recorded at the soma of the 1089 compartment model, with the constituent Tonic, Burst and Quiescent modes labelled. The trimodal repeat length is ~20 s, which is within the experimentally reported range. ***B*** *,* The same trimodal repeat as **(** ***A*** **)** but recorded from a point within the dendritic tree (spiny compartment 1001). One can compare panels **(** ***A*** **)** and **(** ***B*** **)** and observe that with no high-threshold spikes in the dendrites, firing is tonic at the soma. With high-threshold dendritic spikes, bursting occurs at the soma. ***C***, Spontaneous, single trimodal repeat recorded at the soma of the 2 compartment model. ***D***, The same trimodal repeat as panel **(** ***C*** **)**, but recorded from the dendrite compartment of the 2 compartment model. ***E***, Tonic spiking, at the soma, of the full model. ***F***, Burst firing, at the soma, of the full model. ***G***, High-threshold Ca^2+^ spiking, at the dendrite (spiny compartment 1001), of the full model. ***H***, Tonic spiking, at the soma, of the reduced model. ***I***, Burst firing, at the soma, of the reduced model. ***J***, High-threshold Ca^2+^ spiking, at the dendrite, of the reduced model. **Panels A-D** are scaled by the first scale bar (30 mV, 5 s); **Panels E-J** are scaled by the second scale bar (40 mV, 300 ms).

The 2 compartment model can replicate the behavior of the full, 1089 compartment model; it can replicate the cerebellar Purkinje cell’s trimodal pattern of spontaneous activity (Figure 3). The caveat to this is that there is not a complete concordance as regards the Burst mode. High-threshold Ca^2+^ spikes in the dendrites produce bursts at the soma; the frequency of dendritic spiking sets the number of spikes in the burst. The higher the dendritic frequency, the less spikes per burst. In the reduced model, as compared to the full model, the frequency of dendritic spiking is slightly less which results in slightly more spikes per burst. This discrepancy could likely be minimized by further tuning. For example, by reducing the length of the reduced model’s dendrite compartment further (and as per the reduction algorithm, making compensatory changes in radius to keep the volume constant): we observe that the shorter the length of the dendrite compartment, the higher the dendritic spiking frequency and the less spikes per burst. However, we are happy with the present degree of concordance achieved. As we shall demonstrate, our reduced model is a powerful tool for capturing and predicting behavior in the full model.

On an Intel core PC (i5), the 2 compartment model took 1.11 seconds of CPU time for 1 second of simulation. So, simulations with this model run at real time (approximately). This is ~414* faster than the 1089 compartment model.

### Alcohol action upon the Reduced Purkinje neuron model

The model has four different Na^+^/K^+^ pump equations [11]. They each capture different aspects of Na^+^/K^+^ pumping and are founded in previously published Na^+^/K^+^ pump descriptions. They are presented in Equations 52, 54, 137 and 139. An increasing proportion of Na^+^/K^+^ pump molecules being blocked by alcohol is replicated in the model by decreasing the model’s four Na^+^/K^+^ pump densities (*d*^*x*^_*pump*_,_*.*_*g*^*x*^_*pump*_, x = s,d ) by arbitrary functions of time, *t* (in seconds):

147 $$ \begin{array}{l}{d}_{pump}^s(t)={d}_{pump}^s(0)-Yt\\ {}{d}_{pump}^d=\left\{\begin{array}{l}{d}_{pump}^d(0)......................\left[t\le 50s\right]\\ {}{d}_{pump}^d(0)-Mt.............\left[t>50s\right]\end{array}\right.\\ {}{g}_{pump}^x=\left\{\begin{array}{l}{g}_{pump}^x(0).....................\left[t\le 50s\right]\\ {}{g}_{pump}^x(0)-Mt.............\left[t>50s\right]\end{array}\right.\end{array} $$

where x = s,d; Y = 0.014 mA cm^−2^ s^−1^ and M = 0.005 mA cm^−2^ s^−1^. “Catch coding” is code of the form: if (y < 0) [y = 0]; it is used to prevent negative values of (d^x^_pump_,_._ g^x^_pump_, x = s,d) from occurring.

**Figure 4:**
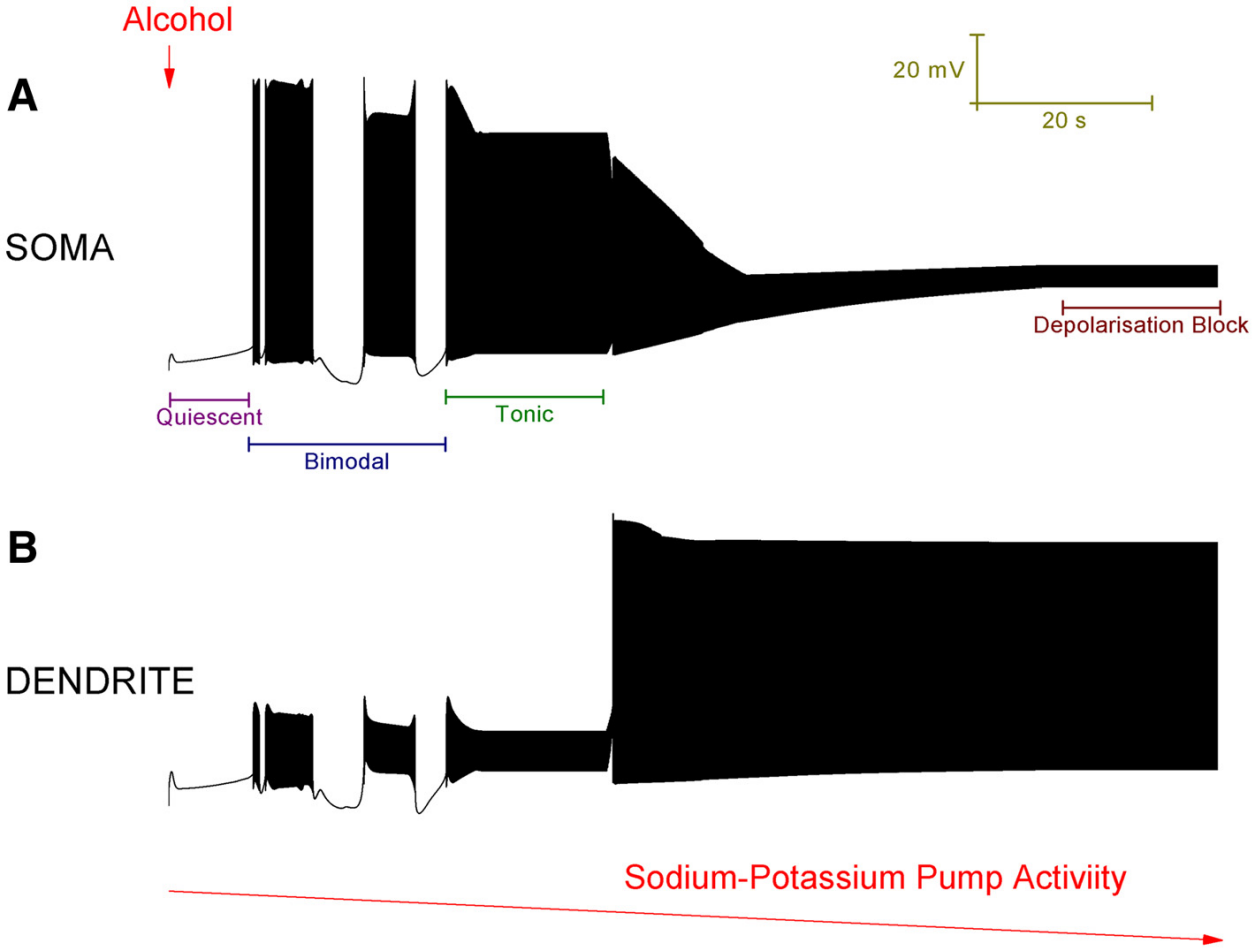
Simulation of alcohol action upon the 2 compartment Purkinje neuron model. ***A***, Membrane potential at the soma. The red arrow, labelled “Alcohol”, symbolises the introduction of alcohol to the model Purkinje cell. A progressing alcohol block of Na^+^/K^+^ pumps is symbolised by the negatively orientated red arrow, labelled “Sodium-Potassium Pump Activity”. As the Na^+^/K^+^ pump activity decreases, the model progresses through a number of labelled activity states: Quiescent, Bimodal, Tonic and Depolarisation Block. Note in the Bimodal phase, the second quiescent period is shorter than the first. ***B***, Membrane potential at the dendrite. Note that during the Depolarisation Block phase at the soma, the dendrites are *not* in depolarisation block and continue to fire. These dendritic spikes travel to the soma and “furrow” its depolarisation block quiescence with small perturbations in membrane potential.

If K_Na_ (Eq. 52) is changed from 40 to 12, which is still a realistic value [20], then the model Purkinje cell is switched from a trimodal pattern of spontaneous firing into quiescence. In experimental recordings, some Purkinje cells are quiescent [13]. From the model’s quiescent state, as the density of the Na^+^/K^+^ pumps is reduced to replicate a progressing alcohol block, then we can observe the Purkinje cell model progress through a number of activity states (Figure 4). These model states correspond to experimentally observed Purkinje cell states [13]. Initially, as the Na^+^/K^+^ pump activity declines, the model cell switches from quiescence into a bimodal pattern repeat of tonic spiking and quiescence. Experimentally, alcohol has been observed to drive some quiescent Purkinje cells into this oscillating pattern [17]. During this mode, observe that the quiescent period gets shorter as Na^+^/K^+^ pump capacity decreases. Eventually, there is not enough Na^+^/K^+^ pump capacity to produce any quiescent periods and the bimodal pattern is replaced by continuous tonic firing. Experimentally, alcohol has been observed to switch some oscillating Purkinje cells into this continuous firing pattern [13]. As the model’s Na^+^/K^+^ pump activity declines further still, the continuous firing pattern is replaced with depolarisation block quiescence. Experimentally, alcohol has been observed to switch some Purkinje cells into this state [13]. With a match to experimental data [13], the model’s depolarisation block state is furrowed with small deflections in the membrane potential. These are attributable to Ca^2+^ spikes that have travelled from the dendrites; although the soma is in depolarization block the dendrites continue to fire.

### Alcohol action upon the Detailed Purkinje neuron model

**Figure 5:**
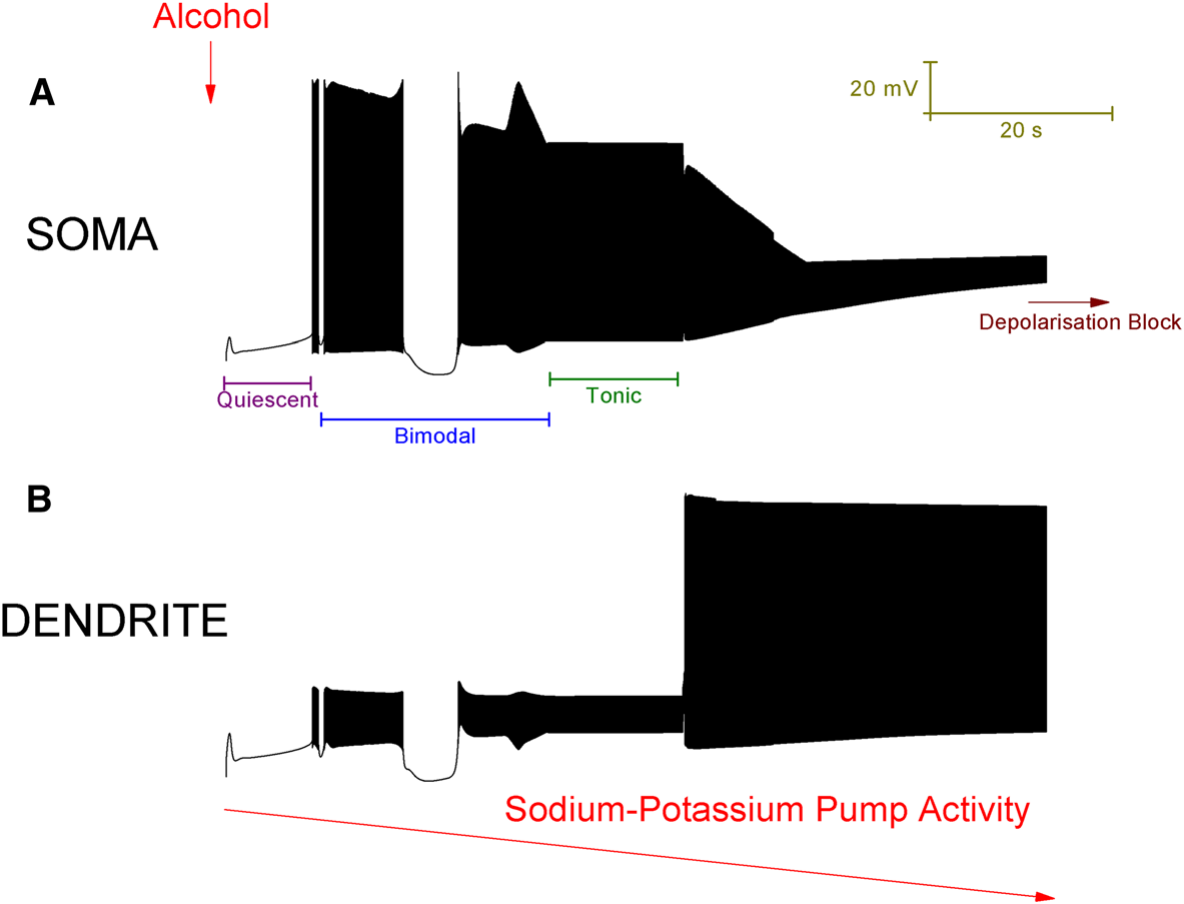
Simulation of alcohol action upon the 1089 compartment Purkinje neuron model. ***A***, Membrane potential at the soma. The red arrow, labelled “Alcohol”, symbolises the introduction of alcohol to the model Purkinje cell. A progressing alcohol block of Na^+^/K^+^ pumps is symbolised by the negatively orientated red arrow, labelled “Sodium-Potassium Pump Activity”. The model progresses through activity states: Quiescent, Bimodal, Tonic and Depolarisation Block. Only the onset of a depolarisation block can be observed because the simulation terminates at 90 seconds (because of issues with the computational expense of the model; refer *Results*). ***B***, Membrane potential at the dendrite (spiny compartment 1001).

As in the reduced model, K_Na_ is changed from 40 to 12 and - as in the reduced model - this switches the model out of an intrinsic propensity to fire in the trimodal firing pattern and into quiescence. To replicate a progressing alcohol block of Na^+^/K^+^ pumps, the model’s four Na^+^/K^+^ pump densities are decreased by the same arbitrary functions of time as in the reduced model (Eq. 147). From the model’s initial quiescent state, as the density of the Na^+^/K^+^ pumps is reduced to replicate a progressing alcohol block, then we can observe the Purkinje cell model progress through a number of activity states (Figure 5). These activity states are the same as those observed, and aforementioned, for the reduced model: Quiescent, Bimodal, Tonic and Depolarisation Block. The only significant difference is that during the reduced model’s bimodal phase, two quiescent periods are observed, but in the full model’s bimodal phase only one quiescent period is observed.

One will note that the reduced model’s response to alcohol is simulated for 120 seconds, whilst the full model’s response to alcohol is only simulated for 90 seconds. This is because of the computational expense of the full model. The reduced model could be run quickly on a laptop (Intel I5) and simulating it for 120 model seconds was not a problem. The full model, however, could not be simulated over this time frame, on these means, within a reasonable time span. We found that this is because the real time taken to simulate a given unit of model time increases to larger values with longer simulations. We simulated the full model for 20 seconds and using the real time taken for this simulation, we extrapolated that a simulation of 120 seconds would take at least 3 days; if not a lot more. The full model was uploaded to the Neuroscience Gateway Portal [46]; RRID: nlx_151553] and run on its Trestles high performance computing (HPC) system, hosted at the San Diego Supercomputer Centre (SDSC). The maximum run time permitted for a job submitted to this system is 15 hours. However, even 15 hours is not long enough for the full model to simulate 120 seconds; only ~90 seconds can be simulated before the 15 hour limit cuts out the simulation. The reduced model runs 560 times faster on a laptop than the detailed model runs on a supercomputer. Furthermore, the former is without the hassles of uploading, job cues, downtime and allocations/limits on use. Investigating the reduced model locally can prime investigations with the full model run on remote resources. Reasonably, we would not have been able to “find” the arbitrary functions of Na^+^/K^+^ pump density decline (Eq. 147) that produce the illustrative behavior in the full model (Figure 5), without going through the intermediary of the reduced model. This point is amplified upon, with another example, in the next section.

Our detailed Purkinje neuron model was run serially on the Trestles supercomputer but we anticipate that our source code could be changed in the future such that it could be run in a parallel fashion. Recently, a parallel computation method has been shown to be viable for single neuron models [47,48] and this method is available on the Neuroscience Gateway Portal [46].

### The two compartment model as a resource for manual tuning of model parameters in a full model

Here we shall demonstrate that the 2 compartment, reduced model can be used as a proxy for tuning the detailed, morphologically faithful model. We show that the reduced model can be used to find parameter values that will permit the full model to replicate new behaviour; that observed experimentally for cerebellar Purkinje neurons in mice that have a BK channel genetic knockout [31,32].

**Figure 6:**
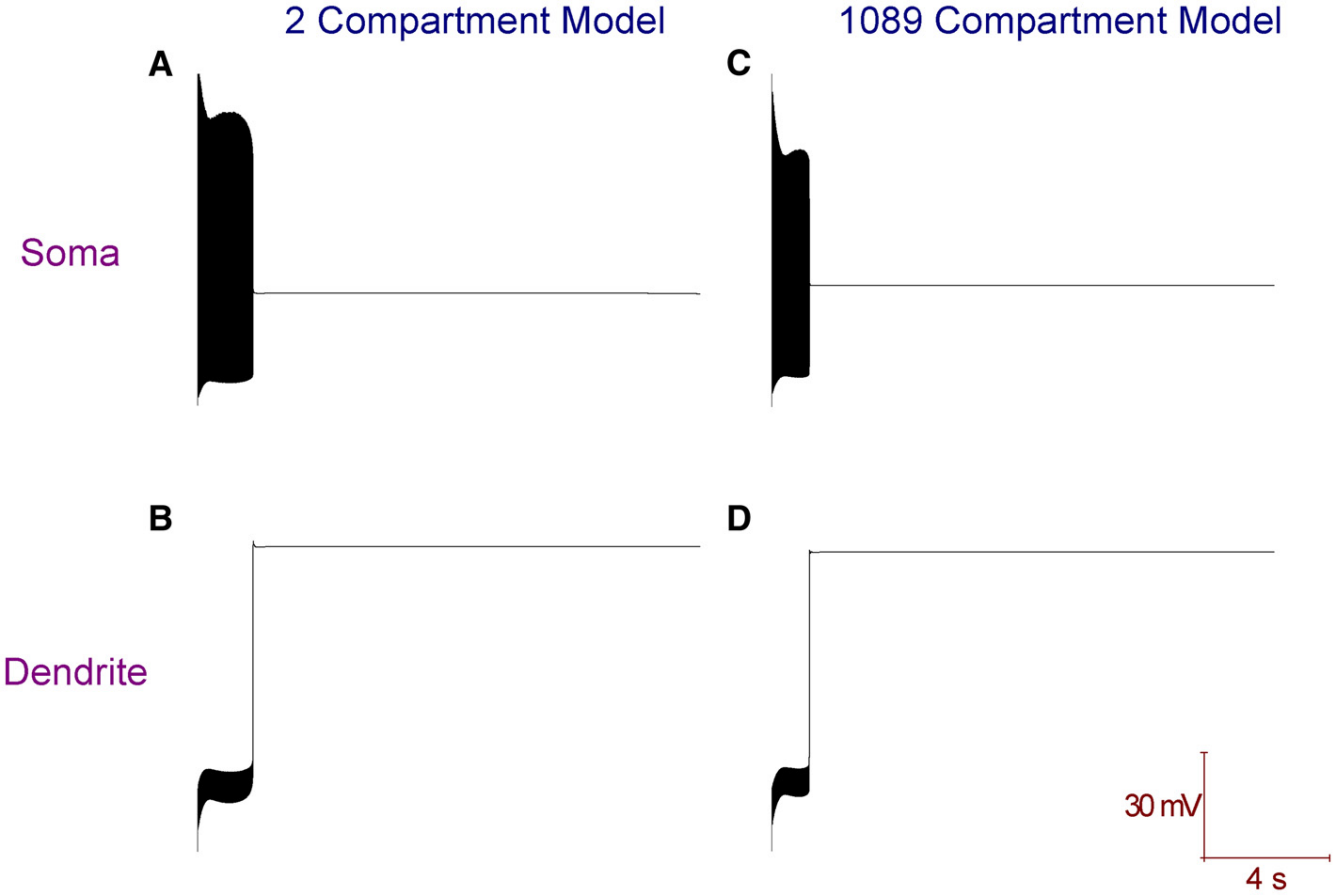
Removal of the BK current (to simulate a genetic knockout of the BK channel) switches both the full and reduced Purkinje neuron models into depolarisation block quiescence. ***A***, Membrane potential at the soma of the 2 compartment model. ***B***, Membrane potential at the dendrite of the 2 compartment model. ***C***, Membrane potential at the soma of the 1089 compartment model. ***D***, Membrane potential at the dendrite of the 1089 compartment model (spiny compartment 1001).

In the full and reduced Purkinje neuron model, Ca^2+^ spikes in the dendrites are repolarised by the BK potassium current. These spikes travel to the soma and generate the bursting mode of the trimodal firing pattern [11]. If this current is removed, the dendrites cannot repolarise after the positive deflection of a Ca^2+^ spike and their membrane potential is then locked at a depolarised value (Figure 6). This depolarisation block in the dendrites then shifts the soma into depolarisation block as well, albeit at a different potential (Figure 6). So, BK removal switches the model cell from a trimodal pattern of spontaneous firing to quiescence (depolarisation block). However, does this correspond to the behaviour of real Purkinje neurons?

**Figure 7:**
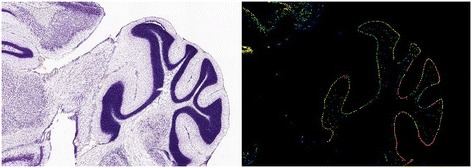
The Allen Brain Atlas (mouse) [53] shows that the β4 auxiliary sub-unit to the BK channel (*KCNMB4*) is expressed in the Purkinje cell layer. The left panel shows a Nissl stained, sagittal slice of the cerebellum from a mouse (Mus Musculus, male, C57BL/6J strain, age: 56). The right panel shows *KCNMB4* expression from this slice using colorimetric in situ hybridisation (RNA probe, antisense, Probe RP_050331_03_G01). This expression aligns with the Purkinje cell layer.

*In vitro*, pharmacological block of BK, by IBTX [7,49,50] or CBTX [51], does not switch Purkinje neurons out of the trimodal firing pattern. However, the BK current in Purkinje cells has been reported to have an IBTX/CBTX-resistant component [52]. The β4 (*KCNMB4*) auxiliary sub-unit confers IBTX/CBTX resistance to BK and this is expressed in the Purkinje cell layer (Allen Brain Atlas, mouse [53]; RRID: nlx_151358], Figure 7).

In the cerebellum of a BK knockout mouse (BK-/-), where BK loss is certainly absolute, a large percentage (>50%) of Purkinje neurons are observed to be stuck in depolarisation block quiescence [31,32]. This is in contrast to the wild-type, where none of them are. This data supports our model. However, even in BK-/- mice, some Purkinje neurons are still observed to fire in tonic and/or burst forms. In this case there must be an alternative current acting to repolarise dendritic Ca^2+^ spikes. This may be due to a “homeostatic remodelling” process [54] – the increased expression of other K^+^ currents during development to compensate for the absence of BK channels. Or it could be that a certain percentage of Purkinje neurons, even in the wild-type, do not use BK to repolarise dendritic Ca^2+^ spikes; but instead use some alternative. So, in different Purkinje neurons, there may be different ionic solutions to producing a certain firing pattern. This may be a more general feature of neurons – there may not be a single, stereotypical set of conductances for a particular neuron type, but a family of different settings – possibly all producing very similar firing characteristics [55].

What alternative conductance could repolarise dendritic Ca^2+^ spikes in Purkinje neurons? Purkinje neurons express Kv3.3 and Kv3.4 in their soma and dendrites [56]. McKay & Turner [51] concluded that these are the principal agents to the repolarisation of dendritic Ca^2+^ spikes. They came to this conclusion because TEA, which blocks Kv3 and BK, produced a much greater deleterious effect upon this repolarisation than CBTX, which blocks BK only. However, as aforementioned, BK in Purkinje neurons has a CBTX resistant fraction and this was not factored into their interpretation. Furthermore, in Kv3.3-/- knockout mice, there is a maintained repolarisation of dendritic Ca^2+^ spikes in Purkinje neurons [57]. Albeit that they are larger (reflecting a diminished repolarisation capacity) and occur more readily (have a lower threshold). SK may be involved but its block by apamin or scyllatoxin does not block repolarisation of dendritic Ca^2+^ spikes and the expression of the trimodal firing motif [6,7]. Intermediate conductance Ca^2+^-activated K^+^ channels (KCa3) are present in Purkinje neurons [58] but this channel class is blocked by CBTX and CBTX application does not block dendritic repolarisation and the trimodal pattern of firing [51].

**Figure 8:**
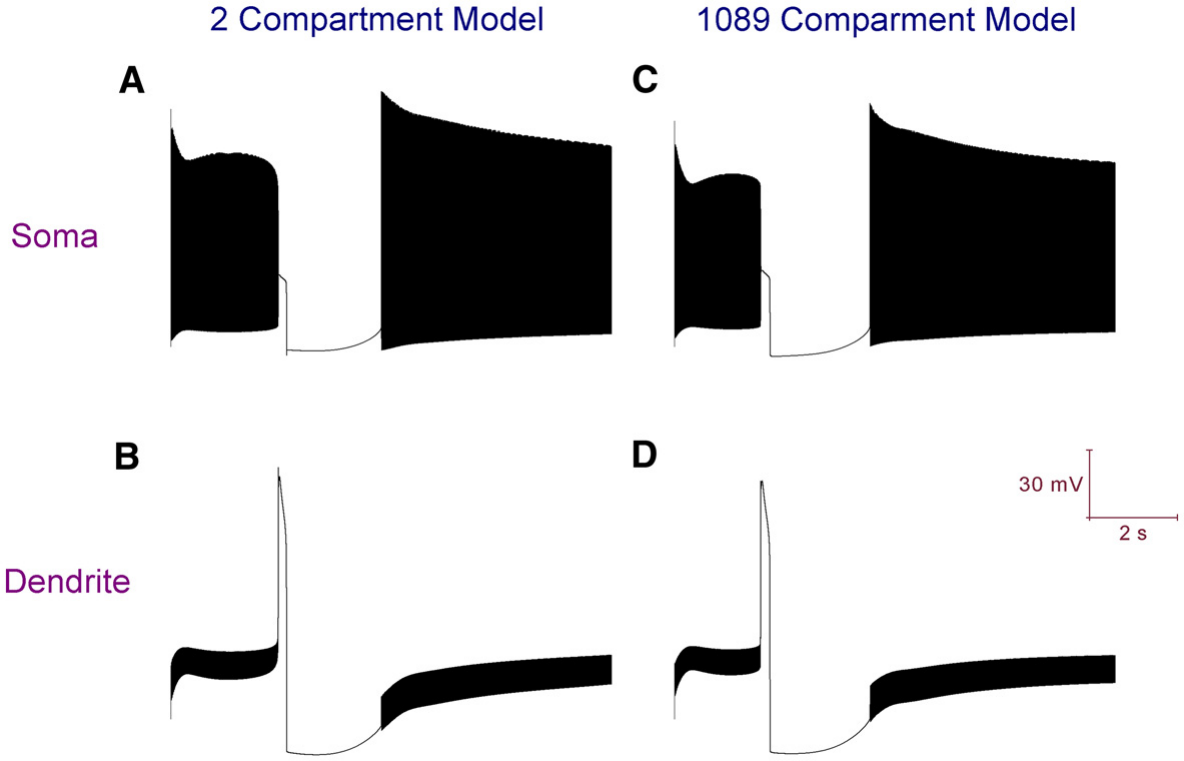
In the full and reduced Purkinje neuron models, an introduced ERG potassium current can repolarise dendritic spikes in the absence of the BK potassium current. BK density is set to 0 in all compartments and ERG is introduced into the dendrite compartments, at an equivalent density (refer Results) for the reduced and full model. ***A***, Membrane potential at the soma of the 2 compartment model. ***B***, Membrane potential at the dendrite of the 2 compartment model. ***C***, Membrane potential at the soma of the 1089 compartment model. ***D***, Membrane potential at the dendrite of the 1089 compartment model (spiny compartment 1001).

Purkinje neurons express an ERG K^+^ current [59] and we suggest that it can repolarise dendritic Ca^2+^ spikes. This hypothesis is founded upon *in silico* experiments with our reduced 2 compartment Purkinje model, in which we demonstrate that an ERG K^+^ current description, sourced from [60], can repolarise our model’s dendritic membrane potential in the absence of BK (Figure 8). The BK maximal conductance is set to 0 in both the soma and dendrite compartments; ERG is introduced into the dendrite compartment with a maximal conductance (g_max_) of (0.5 * *C*_d_) mS/cm^2^, where *C*_d_ is a scaling factor applied to maximal conductance values in the dendrite compartment (refer *Methods*). The ERG current description is from [60], but with the V_half_ value for the *m* activation gate changed from -35 mV to – 5 mV. ERG activation, but not inactivation, is allosterically regulated by external Ca^2+^ to the cell and this updated value is more appropriate for physiological concentrations of external Ca^2+^ [59,60].

148 $$ {I}_{ERG}={g}_{\max}\cdot m\cdot h\cdot \left(V-{E}_K\right) $$

149 $$ {m}_{\infty }=\frac{1}{1+ \exp \left(\frac{-\left[V-{V}_{half}\right]}{5}\right)} $$

150 $$ {h}_{\infty }=\frac{1}{1+ \exp \left(\frac{-\left[V--70\right]}{-20}\right)} $$

151 $$ {\tau}_m=\frac{1}{\left[0.00225* \exp \left(0.12*V\right)\right]+\left[0.00004* \exp \left(-0.05*V\right)\right]} $$

152 $$ {\tau}_h=\frac{1}{\left[0.1* \exp \left(0.02*V\right)\right]+\left[0.003* \exp \left(-0.03*V\right)\right]} $$

Having found that ERG can repolarise dendritic Ca^2+^ spikes in the reduced model, we then tested it with the full model. The BK maximal conductance is set to 0 in all compartments; ERG is introduced into the model’s 1088 dendrite compartments with a maximal conductance (g_max_) of 0.5 mS/cm^2^. We found that ERG can repolarise dendritic Ca^2+^ spikes in the full model also, at an equivalent maximal conductance value (Figure 8). This is the important point to take away here, over and above our illustrative “ERG hypothesis”: That the reduced model can be used as a proxy for tuning of the detailed model.

This result with ERG is intriguing because ERG is not typically associated with roles in the nervous system. Transcripts encoding ERG potassium channels are expressed by most neurons of the CNS but few ERG currents have actually been recorded in these neurons [57]. ERG is typically known for its role in cardiac repolarisation and its dysfunction is associated with a number of cardiac arrhythmias [61,62].

## Discussion

A number of seminal theories of cerebellar functioning [63,64] have considered its component neurons to be linear summing devices (“integrate and fire”), ignoring their complexity, nonlinearity and computations. This is likely too abstractive. We believe that to understand the cerebellar network, researchers should employ network simulations that incorporate more detailed neuron descriptions. The Purkinje cell model of [11] is an intricate *in silico* experimental preparation. It favors detail over abstraction but this makes it computationally intensive and unsuitable for use in network simulations, with the computational resources that most investigators have available. With this in mind, we have used mathematical transforms to produce a simpler, faithful surrogate. We hope that researchers will utilize this in future cerebellar network studies; and identify how distinctive Purkinje cell behaviors are important to network and system function.

### Wider utility of the reduction algorithm

We anticipate that the algorithm used here, to arrive upon a 2 compartment model of the cerebellar Purkinje cell, can be used to generate 2 compartment models of other neuronal types. Some research groups are aiming to model large neuron circuits, and even the entire brain [65], with morphologically realistic neuron descriptions and physiological numbers of brain cells. They aim to address the computational intensiveness issues by utilizing a supercomputer or a massively parallel CPU cluster. However, even with these resources, computational power will remain a significant limiting step for a long time to come; especially for simulations of brain activity on the scale of seconds or minutes. An interim step could be to assemble brain simulations with two compartment descriptions of neurons; using the kind of description that we have produced here for the cerebellar Purkinje cell.

We believe that our method of producing a two compartment model is superior to other methods that rely on an abstractive, coupling parameter between the two compartments [66]. Our method produces a reduced model with more concordance to the full model, and permits findings from the reduced model to be more readily related to the full model.

### Democratising neuroscience research

Computational neuroscience can be expensive. Many computational approaches are costly, requiring infrastructure (clusters, grids, supercomputers etc.) that typical researchers in the developing world cannot afford. However, we show that it is still possible to compete at the bleeding edge of single neuron modelling with just a PC. Extremely detailed neuron models can be developed on very limited computational resources if a reduced model is used as a proxy for development. This approach hinges upon creating and using a reduced 2 compartment neuron model, with its quick run-time, to explore new hypothesises and parameter settings. Then once an achievement has been attained in this system, investigating if the settings found in the reduced model can produce the same behaviour in the full model. It should be the case. The second confirmatory step can be accelerated by uploading the full model to the Neuroscience Gateway Portal [46]; although it can be very protracted even on these resources. This two-step approach can democratise participation in neuroscience research. Anyone with a PC can successfully participate and progress in developing detailed neuron models. This is important because there are many individuals that don’t have access to expensive computational infrastructure and/or experimental laboratory facilities but seek to contribute to science research.

### A biophysical basis to the operating diversity of the cerebellar Purkinje neuron

Our detailed Purkinje neuron model [11] is not an exact simulacrum of a cerebellar Purkinje neuron and our work is not the final word in Purkinje neuron modelling. We have described our full [11] and reduced models in as much detail as we can with the hope that others can build upon our work as we have built upon the work of others e.g. [40,43,65-69]. Through the research community’s co-operative and iterative building, testing and exploring of models – feeding into, and responding to, experimental investigation - we can assemble a formal, quantitative underpinning to neuroscience [70]. Models are especially useful for making sense of disparate data and explaining why different behaviour is observed in different Purkinje cells and/or in different experiments. In this study, we have used modelling to show how Purkinje cells can exhibit different activity states and how alcohol can shift them between these activity states. This work aligns with our previous study [11] and suggests that at the foundation of the Purkinje cell’s intrinsic multimodality, there is the working of just a single molecular species – the Na^+^/K^+^ pump.

### Alcohol and the cerebellar Purkinje neuron

Alcohol consumption corrupts motor function and this is a significant factor in a large number of accidental injuries and deaths every year. The cerebellum controls motor co-ordination [28]. In previous publications we have suggested that the Na^+^/K^+^ pump controls the intrinsic activity of cerebellar Purkinje neurons [11] and that it might be directly involved in cerebellar information processing [25]. Here we suggest that alcohol inhibits Na^+^/K^+^ pumping in cerebellar Purkinje neurons. This may be a factor in the motor dysfunction concordant with inebriation. It would be interesting to use animal models to explore if factors that potentiate Na^+^/K^+^ pumping can counter alcohol associated motor dysfunction. Alcohol may inhibit a proportion of Na^+^/K^+^ pumps but if the activity of the remaining, uninhibited cohort can be increased then this may compensate. This avenue of research could provide one dimension to a “sobriety pill”, taken to counteract the effects of ingested alcohol when they are no longer desired. It would likely not be the complete solution as alcohol probably actions against a number of molecular targets, across numerous brain regions, not simply the Na^+^/K^+^ pump in cerebellar Purkinje neurons [71-91]. However, it may well improve motor co-ordination and reaction times in individuals with alcohol in their system; this may confer some safety benefits. Indeed, more generally, alcohol’s corruptive effect to the motor system is widely considered an unwanted side-effect to alcohol’s coveted effects on mood and sociability. An alcoholic beverage ingredient that could counteract/block alcohol’s effect upon the motor system, and leave its other physiological effects intact, would likely have a commercial potential. However, this aim is complicated because the cerebellum may control some higher-order cognitive and emotional functions, in addition to its motor role [92,93], and may confer additional aspects to the alcohol response. Furthermore, alcohol may act upon other brain cells/regions by Na^+^/K^+^ pump inhibition. Alcohol has been shown to inhibit the Na^+^/K^+^ pump in cerebellar Golgi cells and this could be another locus by which alcohol disrupts cerebellar computation [92-96]. This locus might also be helped by the application of Na^+^/K^+^ pump potentiating agents. Dimethyl sulfoxide (DMSO) has been shown to directly promote Na^+^/K^+^ pump activity [97], is licensed by the FDA as a therapeutic for a number of disorders, is used widely as a dietary supplement but has also been associated with toxicity and side effects e.g. [97]. Follistatin-like 1 is a protein produced by the FSTL1 gene and it can bind to, and increase pumping by, the α1 Na^+^/K^+^ pump isoform [98]. The Allen Brain Atlas [53] shows that FSTL1 is expressed widely in the mouse brain but *not* in the cerebellar cortex (data not shown). If this gene is expressed in this part of the brain, through an experimental intervention, it may have effects upon the behavioural response to alcohol. Although, perhaps not: Purkinje neurons may not express the α1 isoform [99]. Purkinje neurons are particularly rich in the α3 Na^+^/K^+^ pump isoform and Carbon Monoxide (CO) gas, which is likely an endogenous signalling molecule [100], has been shown to increase pumping by this isoform in this cell class [101]. CO seems to promote Na^+^/K^+^ pumping through cGMP and Protein Kinase G (PKG); CO levels are regulated by glutamate binding to glutamate receptors [101]. It might be that this system could be pharmacologically manipulated to counterbalance the effects of alcohol upon cerebellar Purkinje neurons. We now have a crystal structure of the Na^+^/K^+^ pump, and an increasing knowledge of how it functions as we gain atomic resolution views of it in different conformational states [102-107]. We envisage that in the future it will be possible to rationally design new drugs to bind/ interact with it and to directly promote its action. Although, there are already agents, some endogenous, that can fulfil this action e.g. phosphatidylcholine or phosphatidylethanolamine [108]. Alcohol may act upon the Na^+^/K^+^ pump by altering the membrane lipid bilayer in which it floats, which is a factor in setting its structure (hence function), and/or it may interact with the Na^+^/K^+^ pump molecule directly. If the latter, in the future it may be possible to design a drug – using molecular dynamics (MD) simulations - that would interact with the Na^+^/K^+^ pump and block alcohol’s negative action upon it.

### Future work

In this study, we have explored intrinsic electrical activities. The next step would be to simulate and explore the Purkinje cell models under conditions of synaptic input. The Purkinje neuron receives numerous inputs (e.g. ~200,000 parallel fiber (PF) inputs) and it is computationally expensive to simulate them all. Typically, Purkinje modellers only simulate a fraction of the total and can increase the frequency of those modelled, over and above the physiological rate, to compensate for this omission [109,110]. For example, the 200,000 asynchronous PF inputs – each with a firing frequency of ~0.01 Hz - could be approximated by a single PF input with a firing frequency of 2000 Hz; for computational brevity. Our reduced Purkinje cell model is unlikely to be the most minimal biophysical description that can reproduce the dynamical features of the full model. It can probably be further idealized. Although we have reduced the compartment number, we have not investigated if any currents can be omitted. It is likely that some can.

## Conclusions

At present, neuroscience research is heavily reductionist. For example, cellular and system studies are not well integrated. To elucidate brain functioning we need to build bridges between the different levels of description: to relate genes to molecules, molecules to cells, cells to circuits, circuits to systems and systems to perception/behavior. We lay the framework for one such bridge in this study. We produce a reduced Purkinje neuron model with significant cellular fidelity, low computational overhead and suitability for inclusion in large circuit and network simulations. Furthermore, the research of this study reaffirms our previous studies [11,23-27], which assert that the Na^+^/K^+^ pump controls the activity mode of cerebellar Purkinje neurons and is a computational element in the cerebellum. In this paper, we go further and show that alcohol may inhibit the Na^+^/K^+^ pump in cerebellar Purkinje neurons and we propose that this may be the mechanism to alcohol’s corruption of motor control. We speculate that a pharmacological intervention at the Na^+^/K^+^ pump will modulate the body’s physiological response to alcohol.

## Availability of supporting data

Our full (1089 compartment) and reduced (2 compartment) Purkinje neuron models are available in the ModelDB [12; RRID: nif-0000-00004] at http://modeldb.yale.edu/180789.
